# NARFL Knockout Triggers Ferroptosis‐Driven Vascular Endothelial Dysfunction

**DOI:** 10.1002/advs.202415580

**Published:** 2025-11-30

**Authors:** Hui Hu, Jing Luo, Li Yu, Daoxi Qi, Boyu Li, Yating Chen, Chen Wang, Xiaokang Zhang, Wenzheng Guo, Qiyong Lou, Gang Zhai, Yonglin Ruan, Jianfei Huang, Shengchi Shi, Zhan Yin, Fang Zheng

**Affiliations:** ^1^ Center for Gene Diagnosis and Department of Clinical Laboratory Medicine Zhongnan Hospital of Wuhan University Wuhan Hubei 430071 China; ^2^ Department of Laboratory Medicine Shanghai East Hospital Tongji University School of Medicine Shanghai 200123 China; ^3^ State Key Laboratory of Freshwater Ecology and Biotechnology Institute of Hydrobiology Chinese Academy of Sciences Wuhan Hubei 430072 China

**Keywords:** ferroptosis, gene polymorphism, NARFL, oxidative damage, vascular endothelial dysfunction

## Abstract

Nuclear prelamin A recognition factor‐like (NARFL) is a core component of the cytosolic iron‐sulfur (Fe‐S) protein assembly (CIA) system. Yet, its role in vascular pathophysiology remains poorly defined. This study demonstrates that NARFL deficiency triggers ferroptosis, leading to severe vascular endothelial dysfunction across species. In zebrafish, narfl knockout causes embryonic lethality, accompanied by neurovascular defects, blood‐brain barrier disruption, and aberrant hemodynamics. Similarly, knockout of the murine ortholog (Ciao3) results in mid‐gestational embryonic lethality due to impaired vascular development and endothelial progenitor cell maturation. Mechanistically, NARFL deficiency disrupts CIA complex assembly, compromising Fe‐S cluster transfer to client apo‐proteins and leading to a functional shift of cytosolic aconitase (ACO) to iron regulatory protein 1 (IRP1), causing iron overload, heightened oxidative stress, and lipid peroxidation. Consequently, key anti‐ferroptotic defenses are suppressed, culminating in endothelial ferroptosis. This pathway is conserved in human endothelial cells, where NARFL deficiency recapitulates the ferroptotic phenotype and functional impairments, which are rescued by the ferroptosis inhibitor. Clinically, specific NARFL polymorphisms have been identified as conferring susceptibility to vascular endothelial disorders (pulmonary hypertension, epilepsy, and neurodegenerative diseases). The work unveils a novel CIA‐ferroptosis‐vascular axis, positioning NARFL as a critical guardian of endothelial health and a potential therapeutic target.

## Graphical abstract

NARFL is vital for CIA and oxidative stress resistance. NARFL deletion in HPMEC cells, zebrafish, and mice is lethal and rescued by a Ferroptosis inhibitor. NARFL deficiency disrupted its interaction with CIA proteins, decreased aconitase activity, increased IRP1 activity, induced Fe overload, and led to ferroptosis and oxidative stress, resulting in embryonic death and endothelial dysfunction, including pulmonary hypertension, etc.

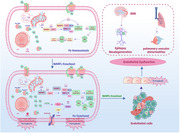

## 1 Introduction

Nuclear prelamin A recognition factor‐like (NARFL) is a critical component of the cytosolic iron‐sulfur protein assembly (CIA) system, which is dedicated to the biogenesis of extra‐mitochondrial iron‐sulfur (Fe‐S) proteins in eukaryotes.^[^ ^1^, ^2^ ^]^ Iron‐sulfur clusters (ISCs) serve as essential cofactors for numerous proteins involved in central cellular processes, including energy and amino acid metabolism, iron homeostasis, DNA replication and repair, and gene expression regulation.^[^ ^3^ ^]^ While mitochondrial ISC biogenesis is well‐characterized, the function and maturation of cytosolic and nuclear Fe‐S proteins remain less explored.^[^ ^3^, ^4^, ^5^ ^]^ The CIA machinery comprises multiple conserved proteins: NUBP1 and NUBP2 form a scaffold for initial ISC transfer, after which NARFL acts as a crucial intermediate carrier.^[^ ^6^, ^7^ ^]^ NARFL subsequently delivers the ISC to the CIA targeting complex (CTC)—composed of CIAO1, MMS19, and MIP18 (CIAO2/FAM96B)—which facilitates its insertion into specific client apoproteins.^[^ ^8^, ^9^ ^]^ Thus, NARFL occupies a pivotal position as an ISC “transmitter” in the CIA pathway, directly influencing the maturation and activity of cytosolic Fe‐S proteins.^[^ ^10^ ^]^

Among the key cytosolic Fe‐S proteins, cytoplasmic aconitase (ACO) is particularly noteworthy for its dual functional role. When equipped with an ISC, ACO catalyzes the conversion of citrate to isocitrate in the tricarboxylic acid cycle.^[^ ^11^ ^]^ However, under low ISC availability, ACO loses its cluster and functions as iron regulatory protein 1 (IRP1), an RNA‐binding protein that post‐transcriptionally controls the expression of genes involved in iron uptake and storage.^[^ ^12^, ^13^ ^]^ This ACO/IRP1 switch represents a fundamental link between cellular iron status and metabolic and redox signaling.

The critical role of NARFL in human disease was highlighted by our recent identification of a novel homozygous mutation, NARFL (NM_022493.3): c.482G>T p. (Ser161Ile), in a rare consanguineous family with pulmonary arterial hypertension secondary to diffuse pulmonary arteriovenous malformations^[^ ^14^ ^]^(Figure S1A‐E, Supporting Information). This mutation was associated with reduced mRNA stability and expression levels of NARFL. Consistent with a pathogenic role, NARFL knockout zebrafish embryos exhibited early lethality and severe vascular malformations, suggesting that NARFL deficiency disrupts vascular development.^[^ ^14^ ^]^

Despite these compelling genetic associations, the precise molecular mechanism by which NARFL deficiency leads to vascular endothelial dysfunction remains largely unknown. Given its central role in the CIA pathway and the functional importance of clients like ACO/IRP1, we hypothesized that NARFL loss disrupts cytosolic Fe‐S protein maturation, leading to aberrant ACO/IRP1 switching, intracellular iron dysregulation, and ultimately, endothelial cell death via ferroptosis—a recently characterized iron‐dependent form of regulated cell death. To test this hypothesis, we employed a multi‐model approach, combining zebrafish and mouse models, human endothelial cell studies, and clinical genetic association analyses. This study aims to elucidate the mechanistic link between NARFL knockout, ferroptosis, and vascular endothelial dysfunction, thereby bridging a critical gap between a rare genetic discovery and a novel pathogenic pathway with broader clinical implications.

## 2 Results

### 2.1 Narfl Knockout in Zebrafish Causes Lethality and Neurovascular Defects

**Figure 1:**
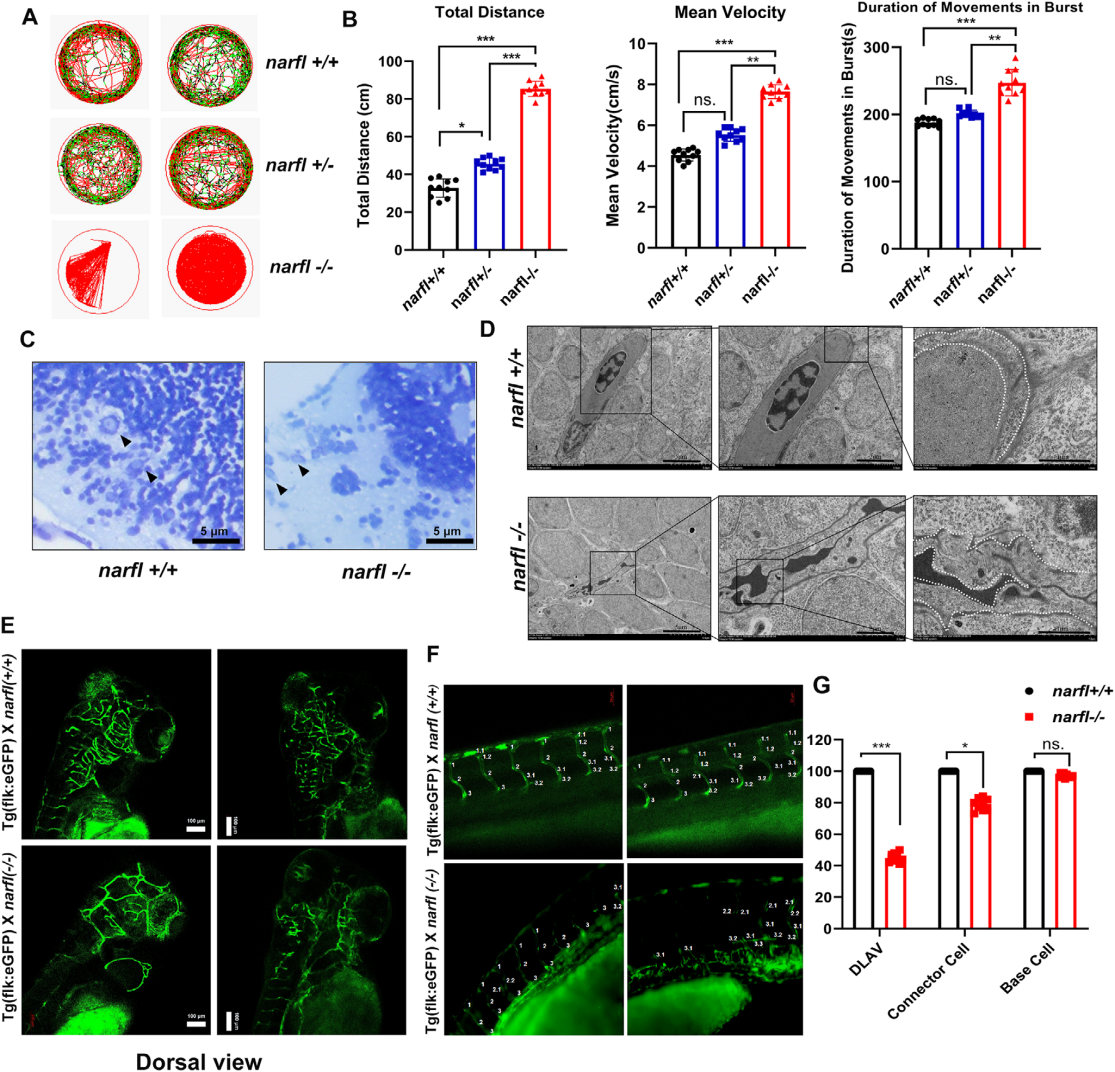
Narfl Deficiency Leads to Abnormal Behavior and Abnormal Blood Vessels and Neurons in Zebrafish. A) Swimming behavior trajectories of zebrafish with different genotypes under normal light. B) Swimming behavior analysis of zebrafish with different genotypes within 60 min under normal light, including total swimming distance, average swimming speed and burst duration analysis (n = 10). C) Toluidine blue staining was used to detect the morphology of Nissle bodies in the brain of zebrafish with different genotypes. D) Transmission electron microscopy (TEM) analysis of the blood‐brain barrier (BBB) ultrastructure in zebrafish revealed distinct morphological differences between wild‐type and *narfl* ^−^ *^/^* ^−^ mutants. In wild‐type zebrafish, endothelial cells appeared plump with no signs of shrinkage, and the basement membrane maintained structural integrity. In contrast, *narfl* ^−^ *^/^* ^−^ zebrafish exhibited pronounced shrinkage of BBB endothelial cells, accompanied by distension and fragmentation of the basement membrane. The field of view of BBB ultrastructure in the brain of zebrafish with different genotypes was observed by transmission electron microscopy (TEM), at 1500×, 5000×, and 10000×, respectively. E) The cerebral vascular morphology of zebrafish with different genotypes was observed by fluorescence inverted microscope imaging system (dorsal view). F) Fluorescence confocal microscope observation and quantitative analysis of zebrafish vascular segments showed that *narfl* ^−^ *^/^* ^−^ dorsal longitudinal anastomosis with blood vessels (DLAV) and the junction cells were obviously absent. G) *narfl* ^−/−^zebrafish dorsal longitudinal cardinal vessels (PCV), connective cells were disordered or even absent, and the structures of dorsal aorta (DA) and PCV were obviously disordered and distorted (n = 5). Statistical significance was assessed using one‐way ANOVA with Tukey's multiple comparison test was used in panels B, F and G. ns, not significant; *p < 0.05; **p < 0.01; ***p < 0.001.

To investigate the physiological consequences of Narfl deficiency at the organismal level, we first characterized the phenotypic abnormalities in zebrafish models. The *narfl* ^−/−^ zebrafish exhibited early lethality, with most mutants dying before 13 days post‐fertilization (dpf) and displaying morphological deformities during development (Figure S2A, Supporting Information). Behavioral analysis revealed hyperactivity, including increased total swimming distance and average speed, alongside spontaneous, irregular movement bursts resembling epileptic seizures^[^ ^15^, ^16^ ^]^ (**Figure** **1**A,B). Neuronal pathology was evident from dissolved and flattened Nissl bodies (Figure 1C). Transmission electron microscopy (TEM) revealed a disrupted blood‐brain barrier (BBB) ultrastructure, featuring shrunken endothelial cells and a fragmented basement membrane (Figure 1D). Confocal imaging of Tg (*flk*:eGFP) zebrafish confirmed severe impairment of cerebral vasculature and a significant reduction in BBB coverage to 32% compared to wild‐type (Figure 1E; Figure S2B, Supporting Information). Vascular analysis further showed disorganized or absent dorsal longitudinal anastomotic vessels (DLAV) and distorted structures of the dorsal aorta (DA) and posterior cardinal vein (PCV) (Figure 1F,G; Figure S2D, Supporting Information). γ‐H2AX detection indicated significant DNA damage in these major vessels (Figure S2E,F, Supporting Information). TUNEL staining showed no significant increase in apoptosis (Figure S2C, Supporting Information).

### 2.2 Narfl Deficiency Disrupts Hemodynamics and Induces a Ferroptotic Phenotype in Zebrafish

**Figure 2:**
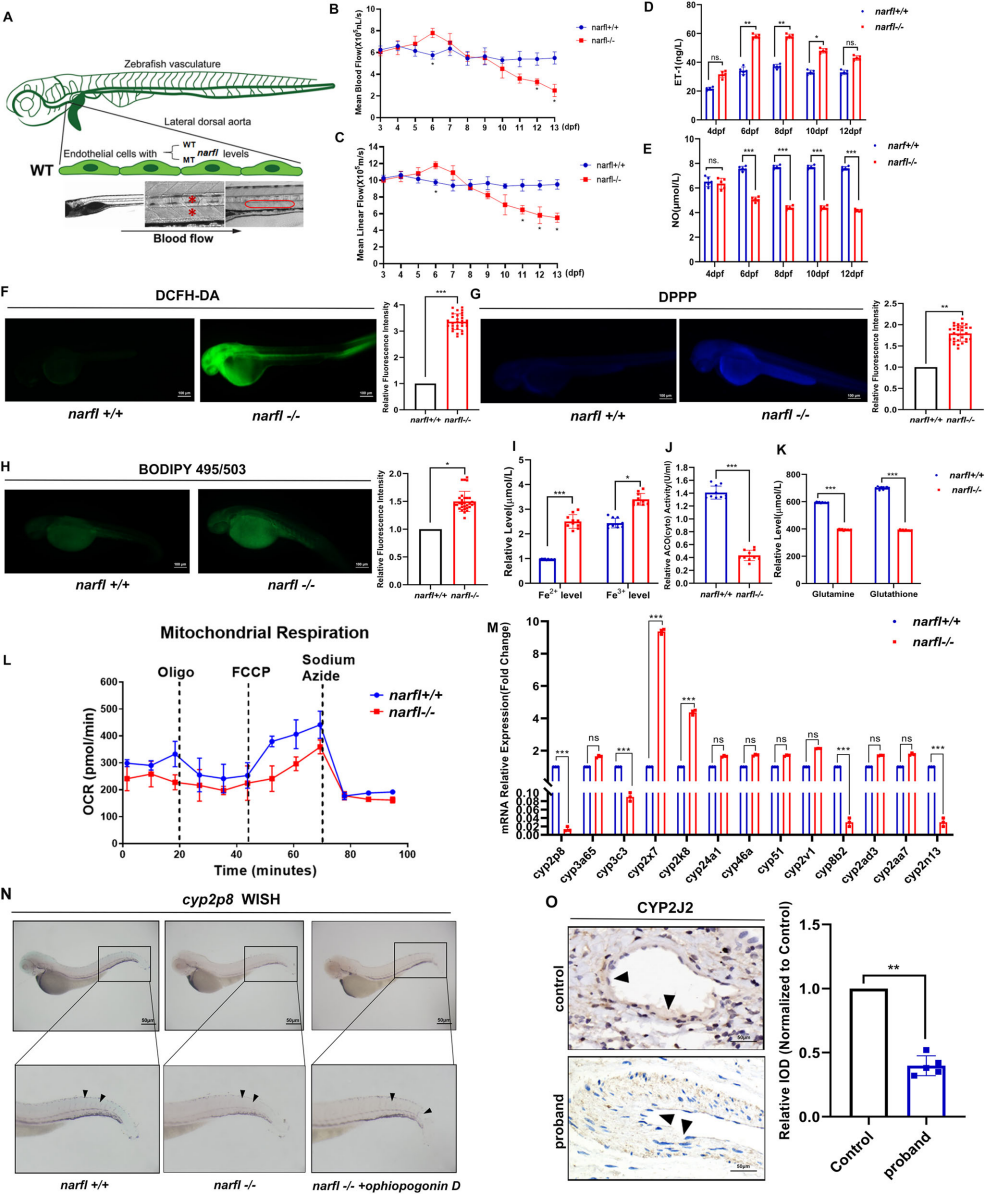
Narfl Deletion Induced zebrafish Endothelial Dysfunction by Upregulating the Iron Level and Production of Reactive Oxygen Species and Lipid Peroxidation. A) The Micro Zebra Lab system detects the blood flow of zebrafish, the red asterisk part detects the line speed, and the red box part detects the average speed. B–E) The mean blood flow velocity (B, D) and mean linear velocity (C, E) of *narfl* ^−/−^zebrafish were not significantly different from those of wild type zebrafish at 3–5 dpf, significantly higher at 6 dpf, significantly lower at 7–11 dpf, and significantly lower at 11–13 dpf than those of wild type (n = 5). F) DCFH‐DA probe was used to detect oxidative stress in 5 dpf zebrafish (n = 30). The stronger the green fluorescence, the higher the oxidative stress level; the magnification was 200×; Compared with wild zebrafish, the *narfl* ^−^ *^/^* ^−^ zebrafish fluorescence intensity was significantly enhanced. G) DPPP probe was used to detect lipid peroxidation levels in 5 dpf zebrafish (n = 30). The stronger the purple fluorescence, the higher the lipid peroxidation level. H) BODIPY 493/503 probe was used to detect neutral lipid levels (n = 30), with stronger green fluorescence indicating higher lipid levels. I) Determination of iron level in zebrafish (n = 5) by colorimetric method; the results showed that the iron level in *narfl* ^−/−^ zebrafish was significantly higher than that in wild type (*p* <0.001), and Fe^3+^ was also increased. J) Quantitative results of detection of cytoplasmic cis‐aconitase activity in zebrafish (n = 5), the results showed that the cytoplasmic ciaconitase activity of *narfl* ^−^ *^/^* ^−^ zebrafish was significantly reduced. K) Quantitative detection of glutathione and glutamine levels in zebrafish. The results showed that glutathione and glutamine levels in *narfl* ^−/−^ zebrafish were significantly reduced. L) The results of mitochondrial respiration integration of 11 wild type and 8 *narfl* ^−/−^ zebrafish in two groups, which indicated that the deletion of the *narfl* gene led to a decrease in mitochondrial respiratory function of zebrafish. M) qRT‐PCR was used to verify the P450 family genes with differences in RNA sequencing, and the results showed that *cyp2p8, cyp3c3, cyp8b2*, and *cyp2n13* were significantly decreased in *narfl* ^−/−^ zebrafish. *cyp2×7, cyp2k8*, and *cyp2v1* were significantly increased, while there was no significant difference in other genes. N) In situ hybridization was used to detect 5 dpf wild type and *narfl* ^−/−^ zebrafish, as well as the results of treatment with CYP2P8‐specific activator ophiopogon D. The locations indicated by the black arrows showed the sites with positive *cyp2p8* probe signals. O) The expression of CYP2J2 in adjacent lung tissue and in the progenitor with diffuse pulmonary malformation was detected by immunohistochemistry, CYP2J2 is expressed in the cytoplasm and decreased expression of CYP2J2 was found in progenitors with down‐regulated NARFL expression. Statistical significance was assessed using one‐way ANOVA with Tukey's multiple comparison correction. ns, not significant; *p < 0.05; **p < 0.01; ***p < 0.001.

**Figure 3:**
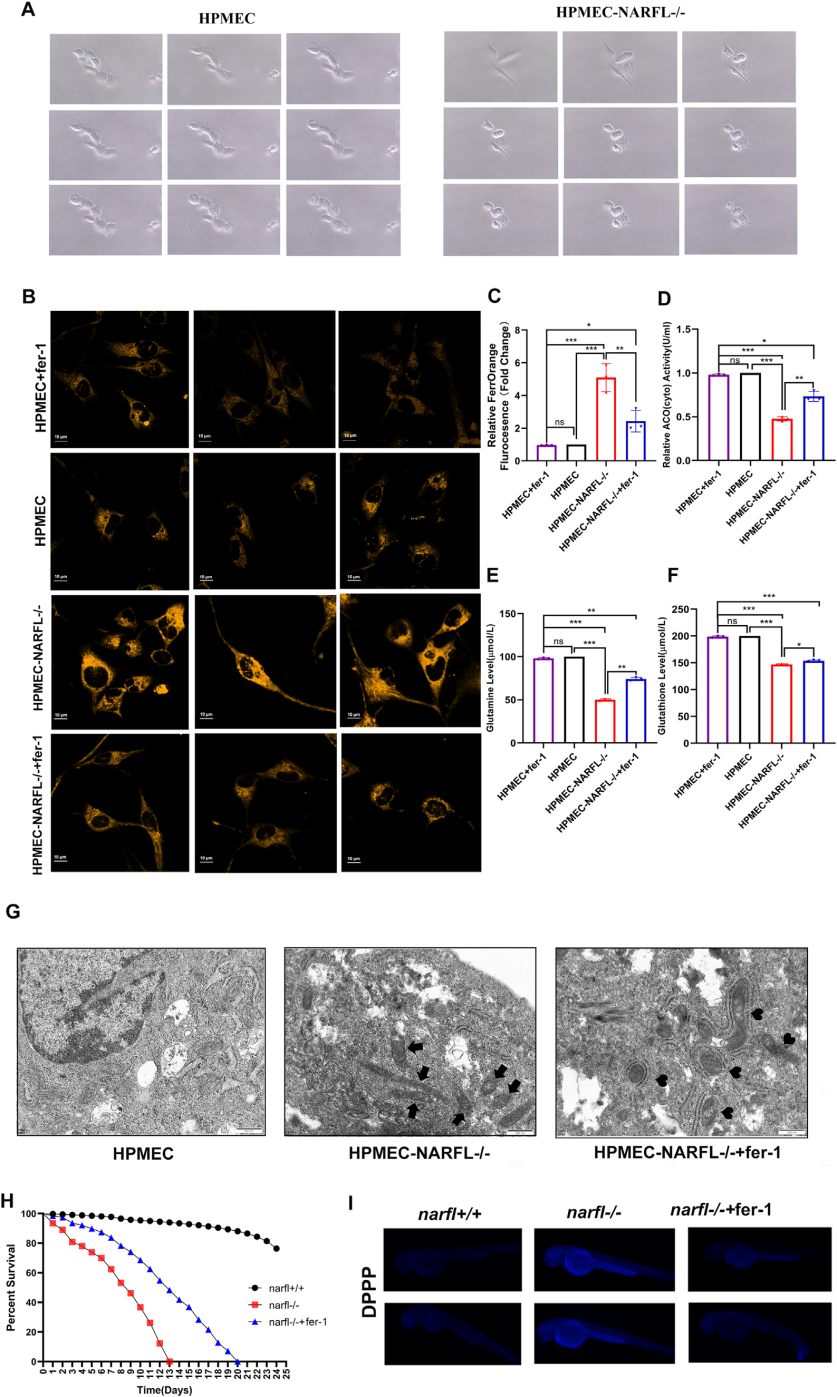
Endothelial Knockdown of NARFL Promotes Ferroptosis and Ferrostatin‐1 Alleviates Oxidative Stress Injury Caused by *NARFL* Gene Deletion. A) Dynamic observation under a microscope and daily photographing of HPMEC wild‐type cells and *NARFL* mutant cells recorded significant differences in cell death patterns between the two groups. *NARFL* mutant cells exhibited clear morphological changes, gradually transitioning to an enlarged and fattened morphology with increased granularity, and their death morphology was similar to that of cells undergoing ferroptosis. B) FerroOrange fluorescence probe was taken by confocal fluorescence microscopy for the detection of HPMEC, NARFL^−/−^ HPMEC, and NARFL^−/−^ HPMEC cells treated with Ferrostatin‐1 (6 µM). The darker orange color indicated the higher content of ferrous ions in the cells. C) Quantitative results were measured by the FerroOrange fluorescent probe; ferrous ion content in HPMEC increased significantly after NARFL deletion, but decreased significantly after Ferrostatin‐1 was added. D‐F) The quantitative results of cytoplasmic cisaconitase (D), glutamine (E) and glutathione (F) in HPMEC, NARFL^−/−^HPMEC and 6 µM Ferrostatin‐1 treated NARFL^−/−^ HPMEC cells, which showed that NARFL deletion can also cause the decrease of cytoplasmic cis‐aconitase activity and intracellular glutathione and glutamine content in endothelial cells, which can be partially saved by ferroptosis inhibitor Ferrostatin‐1. G) Transmission electron microscopy showed that the mitochondrial morphology of NARFL^−/−^HPMEC was obviously different from that of wild type cells. The black arrows indicate mitochondria exhibiting typical ferroptotic morphology, including becoming smaller, increased membrane density, and diminished cristae. H) Survival analysis showed that 8 µM Ferrostatin‐1 can extend the survival time of *narfl* ^−/−^ zebrafish from 13 days to 21 days. I) Detection of 5 dpf zebrafish treated with 8 µM Ferrostatin‐1 by DPPP fluorescent probe; the results showed that 8 µM Ferrostatin‐1 inhibits lipid peroxidation levels caused by narfl deletion. Statistical significance was assessed using one‐way ANOVA with Tukey's multiple comparison correction. ns, not significant; *p < 0.05; **p < 0.01; ***p < 0.001.

Hemodynamic assessment from 3–13 dpf showed an aberrant blood flow pattern in *narfl* ^−^ *^/^* ^−^ zebrafish, characterized by a significant increase in velocity at 6 dpf followed by a sharp decrease thereafter (**Figure** **2**A–C). This was accompanied by endothelial dysfunction, marked by elevated Endothelin‐1 (ET‐1) and reduced nitric oxide (NO) levels from 6 dpf onward (Figure 2D,E). Mutants exhibited a pronounced pro‐ferroptotic state, with significant increases in reactive oxygen species (ROS) (Figure 2F), lipid peroxidation (Figure 2G,H), and intracellular iron levels (Figure 2I; Figure S3B, Supporting Information). This was consistent with reduced cytoplasmic aconitase activity and decreased glutathione/glutamine (GSH‐GL) levels (Figure 2J,K). Mitochondrial respiratory function was significantly impaired (Figure 2L; Figure S3H, Supporting Information). Transcriptomic and qRT‐PCR analyses identified cyp2p8 as the most significantly downregulated gene within the cytochrome P450 family (Figure 2M; Figure S3E, Supporting Information). Whole‐mount in situ hybridization (WISH) confirmed its vascular expression and decrease in mutants, which was slightly rescued by its specific activator, Ophiopogonin D (Figure 2N). This finding was conserved in humans, as immunohistochemistry showed reduced CYP2J2 expression in the lung tissue of a proband with a NARFL mutation (Figure 2O). Ferroptosis inhibitor Ferrostatin‐1 (8 µM) effectively extended the survival of *narfl* ^−^ *^/^* ^−^ zebrafish, reduced lipid peroxidation, and partially restored glutathione levels and cytoplasmic aconitase activity (**Figure** **3**H,I; Figure S3C,D,F,G, Supporting Information). No significant increase in apoptosis was detected by AO staining (Figure S3A, Supporting Information).

### 2.3 NARFL Knockout in Human Endothelial Cells Triggers Ferroptosis and Functional Impairment

**Figure 4:**
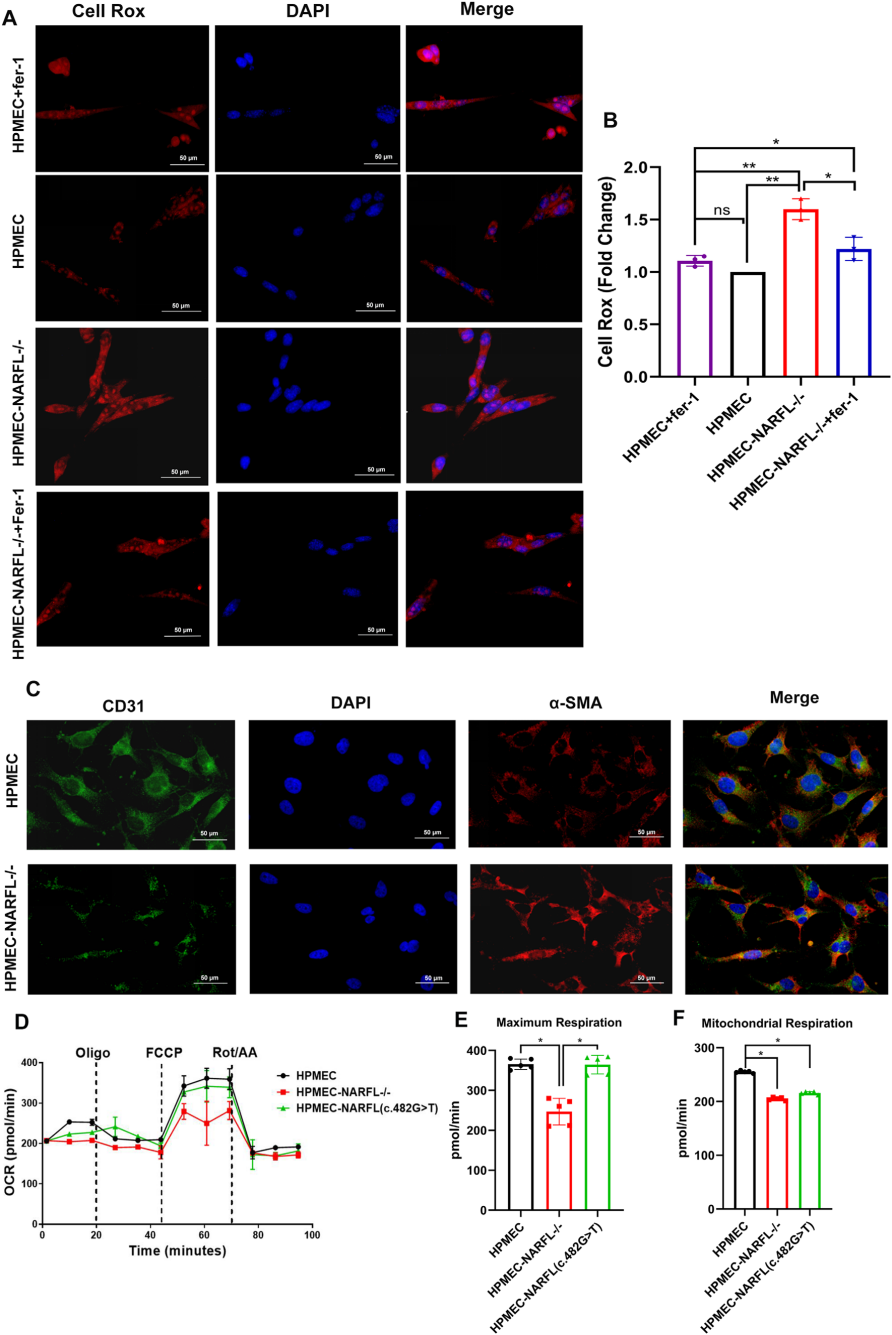
*NARFL* Gene Deletion Leads to Endothelial Cell Dysfunction. A) Cell Rox kit was used to detect the oxidative stress level of endothelial cells. The darker the color of red fluorescence, the higher the level of cellular oxidative stress. B) Quantitative results of cellular oxidative stress were detected by CellRox kit; the results showed that NARFL deletion led to an increase in oxidative stress level of endothelial cells, and ferroptosis inhibitor Ferrostatin‐1 could partially alleviate oxidative stress injury caused by *NARFL* gene deletion. C) The expression levels of endothelial cell marker CD31 and fibroblast marker α‐SMA in HPMEC and NARFL^−/−^ HPMEC were detected by immunofluorescence. The fluorescence intensity directly correlates with protein expression levels, and the result showed that deletion of the *NARFL* gene led to lower CD31 (red) expression and up‐regulation of α‐SMA (green). D‐F) HPMEC, NARFL^−^ *^/^* ^−^ HPMEC, and HPMEC transfected NARFL point mutant (c.482 G> T) mitochondrial respiratory function curve, black represents wild type, green represents NARFL (c.482 G>T) point mutant, with red representing HPMEC with NARFL deletion mutations. The results showed that mitochondrial respiratory function decreased with NARFL (c.482 G>T) point mutant, but there was little difference compared with wild‐type cells, while NARFL deletion mutation resulted in significant decrease of mitochondrial function. Statistical significance was assessed using one‐way ANOVA with Tukey's multiple comparison correction. ns, not significant; *p < 0.05; **p < 0.01; ***p < 0.001.

Immunofluorescence of patient lung tissue showed decreased NARFL and endothelial marker CD31, and increased α‐smooth muscle actin (α‐SMA) (Figure S1F, Supporting Information). We established NARFL‐knockout HPMEC lines (Figure S4A‐D, Supporting Information), which exhibited slow growth (Figure S4E, Supporting Information) and a progressive morphological shift to a ferroptotic‐death phenotype (Figure 3A; Videos S1,S2 Supporting Information). NARFL mutant cells exhibited distinct morphological changes, gradually transitioning to an enlarged and fattened morphology with increased granularity. Their death morphology was similar to that of cells with ferroptosis reported by Jiang Xuejun's team.^[^ ^17^ ^]^ TEM showed that the mitochondrial morphology of wild type cells was obviously different from NARFL mutant cells, whose mitochondria became smaller, mitochondrial membrane density increased significantly, and cristae decreased, which was consistent with the morphology of ferroptosis^[^ ^18^ ^]^ (Figure 3G; Figure S4F, Supporting Information). NARFL^−^ *^/^* ^−^ cells showed increased ferrous iron (Figure 3B,C), decreased cytoplasmic aconitase activity, and reduced GSH‐GL levels, all partially rescued by Ferrostatin‐1 (Figure 3D–F). The knockout cells also showed elevated oxidative stress (**Figure** **4**A,B), and mirrored the patient tissue findings with downregulated CD31 and upregulated α‐SMA, a marker of fibroblasts,^[^ ^19^ ^]^ consistent with the immunohistochemical features of the proband's lung tissue (Figure 4C; Figure S1F, Supporting Information). While a point mutant (NARFL‐c.482G>T) only slightly reduced mitochondrial respiration, full NARFL knockout severely impaired it without affecting glycolysis (Figure 4D–F; Figure S5A‐C, Supporting Information).

**Figure 5:**
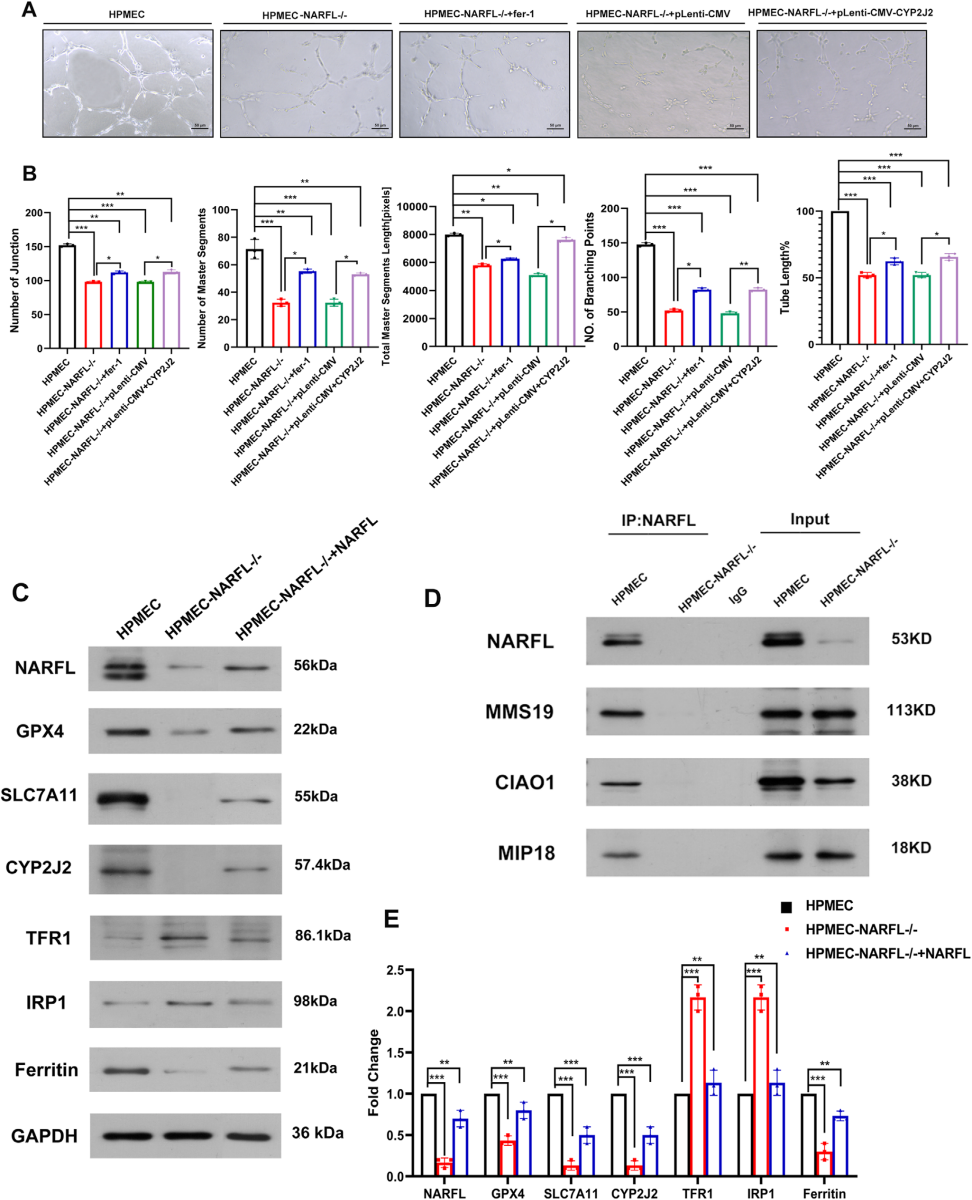
*NARFL* Gene Deletion Leads to Endothelial Cell Dysfunction with Abnormal Ferroptosis Pathway and Inability to Interact with CIA System‐Related Proteins in Endothelial Cells. A,B) Tube formation experiment compared HPMEC, NARFL^−^ *^/^* ^−^ HPMEC, NARFL^−^ *^/^* ^−^ HPMEC treated with Ferrostatin‐1 inhibitor of iron ions, and NARFL^−/−^HPMEC transfected with *CYP2J2* plasmid. The results showed that NARFL deletion would lead to difficulties in tubular formation. However, Ferrostatin‐1 and the overexpression of the *CYP2J2* plasmid can partially alleviate the tubule formation difficulty. C,E) Western blot results of HPMEC wild‐type cells, NARFL^−^ *^/^* ^−^ HPMEC cells, and NARFL^−^ *^/^* ^−^ HPMEC cells transfected with the overexpression of the NARFL plasmid. The results showed that lower NARFL expression led to lower expression of GPX4, SLC7A11 and Ferritin. The expression of TFR1 and IRP1 was up‐regulated, whereas CYP2J2 was barely expressed in NARFL^−^ *^/^* ^−^ HPMEC; transfection with an overexpression NARFL plasmid in NARFL^−^ *^/^* ^−^ HPMEC partially compensated for these changes. D, Result of co‐immunoprecipitation; the Co‐IP result shows the NARFL mutant could not interact with MMS19, CIAO1 and MIP18. Statistical significance was assessed using one‐way ANOVA with Tukey's multiple comparison correction. ns, not significant; *p < 0.05; **p < 0.01; ***p < 0.001.

Sequentially, we compared the tubule formation function and cell osmotic ability among wild‐type HPMECs, NARFL deletion HPMECs, NARFL deletion HPMECs treated with Ferrostain‐1, and NARFL deletion HPMECs transfected with CYP2J2 plasmid (**Figure** **5**A). The results showed that NARFL deletion led to difficulty in tubule formation and reduced cell osmotic ability, but Ferrostain‐1 and CYP2J2 overexpression could partially rescue these effects (Figure 5B; Figure S5D‐F, Supporting Information).

### 2.4 NARFL Deletion Activates the Ferroptosis Pathway and Disrupts the CIA Complex

So far, we have found that NARFL deficiency can lead to increased intracellular iron and oxidative stress, resulting in ferroptosis, but the specific mechanism remains unclear. Intracellular iron metabolism begins with iron bound to transferrin in circulation, which is endocytosed into cells by binding with TFR1 on the cell membrane to form an unstable iron pool, and some of them are stored in the cytoplasm in the form of ferritin. In cells undergoing ferroptosis, the contents of iron and transferrin increase, but the amount of membrane iron transporter decreases^.[^ ^20^ ^]^ GPX4 is the hub molecule regulating ferroptosis,^[^ ^21^ ^]^ which converts GSH into oxidized glutathione and protects cells from ferroptosis by restricting cytotoxic lipid peroxidation. GSH‐peroxidase pathway is further regulated by cystine transporter system Xc^−^ (composed of catalytic subunit SLC7A11 and chaperone subunit SLC3A2). Cystine uptake mediated by SLC7A11 plays a key role in inhibiting oxidative reactions and maintaining cell survival under oxidative stress. By Western blotting, we found that *NARFL* deficiency could lead to reduced expression of GPX4, SLC7A11 and Ferritin, while TFR1 and IRP1 were up‐regulated. In the zebrafish model study, NARFL deletion decreased the expression of iron‐sulfur protein cyp2p8 (CYP2J2 in humans). In the HPMEC cell model, we found that CYP2J2 was barely expressed in NARFL deletion HPMECs. Transfection and overexpression of the *NARFL* plasmid in NARFL deletion HPMECs could compensate for some of the above changes. These results indicated that lower NARFL expression could not only lead to up‐regulation of IRP1, which further upregulated TFR1 and downregulated Ferritin, but also inhibited SLC7A11 and GPX4, thereby activating the ferroptosis pathway (Figure 5C–E). Consistently, it was found that *NARFL* could not bind to CIAO1, MIP18, and MMS19 in HPMECs lacking NARFL, as detected by immunoprecipitation. Probably, since NARFL was first bound to CIAO1, the WB results showed that lower NARFL expression directly decreased CIAO1 expression, while the expression of MMS19 and MIP18 was not affected. Therefore, the deletion of NARFL would lead to the failure of mitochondrial ISCs to be normally transmitted to CIA system‐related proteins, and the failure of ISCs to embed specific client apoproteins, which would eventually affect the synthesis and maturation of cytoplasmic iron‐sulfur proteins (Figure 5D).

### 2.5 Ciao3 Knockout in Mice Confirms Embryonic Lethality and Vascular Dysplasia via Ferroptosis

**Figure 6:**
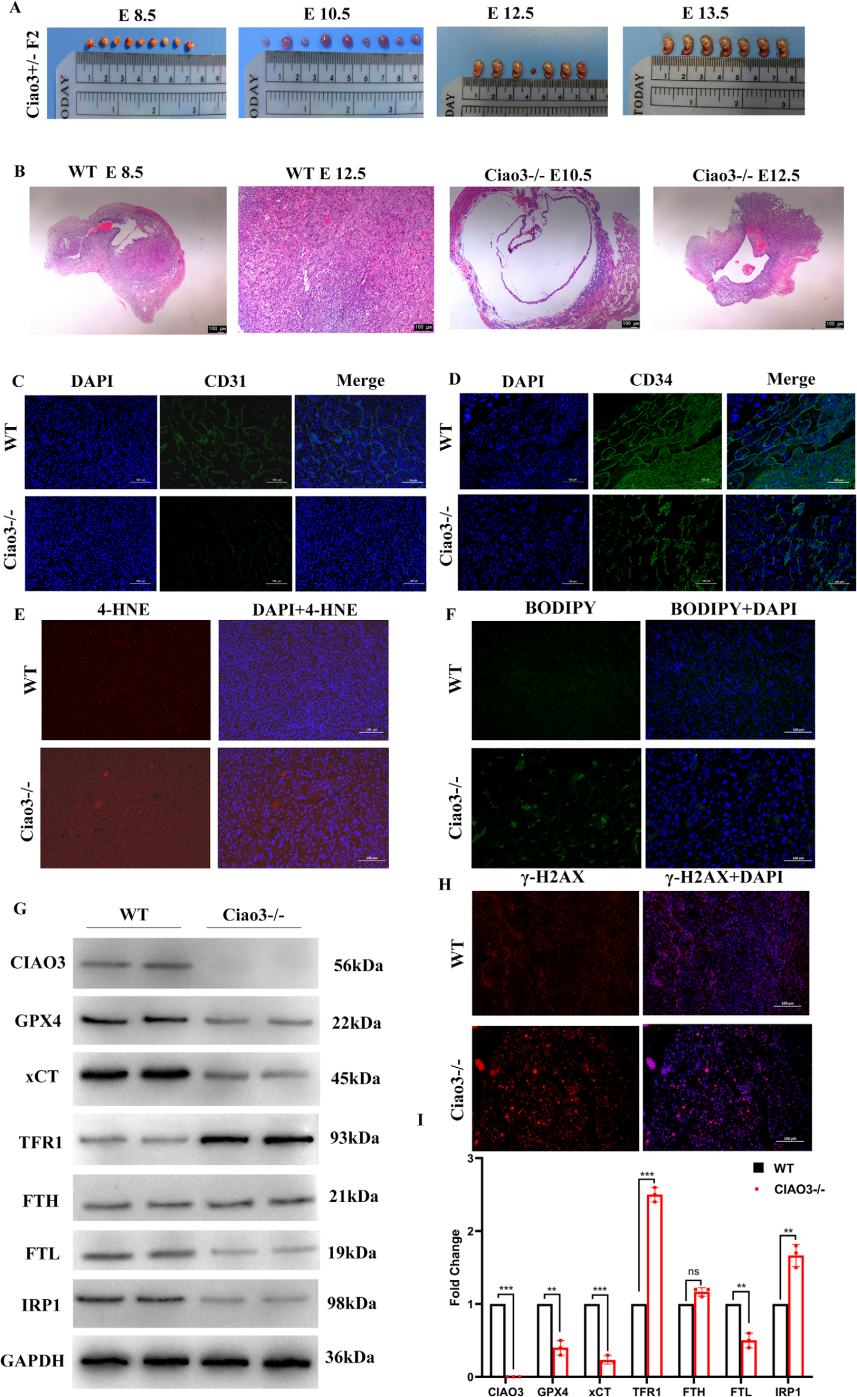
Deletion of the *Ciao3* Gene Leads to Embryonic Death and Vascular Development Disorder in Mice. A) The embryo morphology of the Ciao3 heterozygous offspring at 8.5, 10.5, 12.5 and 13.5 days; Ciao3 knockout embryos still existed at 12.5 days but were completely absorbed at 13.5 days and later. B) H&E staining morphology of wild type with 8.5‐day and 12.5‐day mouse embryos and Ciao3^−/−^ mice embryos with 10.5‐day and 12.5‐day, the development of Ciao3 knock‐out embryos was significantly slower than that of wild‐type mouse embryos and the development of the vascular system was blocked. C) The endothelial marker CD31 was detected by immunofluorescence staining in 12.5‐day mouse embryos. D) The endothelial progenitor cell marker CD34 was detected by immunofluorescence staining in 12.5‐day mouse embryos. E,F) The sections of 12.5‐day‐old mouse embryos were detected by 4‐HNE (E) and BODIPY (F) probes; the positive rate of 4‐HNE and BODIPY in Ciao3^−^ *^/^* ^−^ mouse embryo was significantly higher than in wild‐type mouse embryo. H) γ‐H2AX detection of 12.5‐day mouse embryos showed that the DNA damage of Ciao3^−^ *^/^* ^−^ mouse embryos was significantly enhanced compared with wild‐type embryos. G‐I) Western blotting showed that GPX4, xCT and FTL were significantly down‐regulated in Ciao3 knockout mouse embryos; the expression of TFR1 and IRP1 was significantly up‐regulated. Data were analyzed by an unpaired Student's t‐test; *p < 0.05; **p < 0.01; ***p < 0.001.

To establish conservation of this mechanism in mammals, we characterized Ciao3 (the murine ortholog of human *NARFL*; Gene ID: 123456) knockout mice. As reported by Song et al,^[^ ^10^ ^]^ Ciao3 deletion leads to lethality in the embryo and all embryos of Ciao3 knockout mice were absorbed before 10.5 days, so we took out mouse embryos at 8.5 days, 10.5 days, 12.5 days and 13.5 days respectively and carried out genotype identification, in order to further determine the lethal mechanism of Ciao3 knockout. The results showed that Ciao3 knockout embryos persisted until 12.5 days, whereas they were completely absorbed by 13.5 days and later (**Figure** **6**A). H&E staining of wild‐type embryos and Ciao3 knock‐out embryos showed that the development of Ciao3 knock‐out embryos was significantly slower than that of wild‐type embryos, and the development of the vascular system was blocked. Furthermore, to assess whether the observed vascular patterning defects were linked to impaired endothelial proliferation, we performed EdU (5‐ethynyl‐2′‐deoxyuridine) incorporation assays in conjunction with endothelial marker (Endomucin) staining. As shown in Figure S6B (Supporting Information), Ciao3^−/−^ embryos exhibited a poorly formed and disorganized Endomucin‐positive vascular network, which was accompanied by a marked reduction in EdU incorporation compared to wild‐type littermates. This indicates that the deletion of Ciao3 concurrently impairs both vascular patterning and endothelial cell proliferation, contributing to the embryonic lethal phenotype. Heterozygotes of Ciao3 knock‐out mice were born normally, and there was no significant difference in phenotypes between them and wild type mice. Blood vessels in the yolk sac of homozygous Ciao3 knock‐out mice became thinner, branches decreased, the vascular network was incomplete, compactness decreased, and vascular development was blocked (Figure 6B). So, we hypothesized that abnormal vascular development might be the cause of death of Ciao3 knock‐out mice. During embryogenesis, endothelial cells participate in the development of the cardiovascular system and originate from blood islands, which are derived from mesoderm. To validate our hypothesis, endothelial progenitor cell markers CD31 (Figure 6C) and CD34 (Figure 6D) were detected in whole embryos by immunofluorescence. The results showed that the vascular structure in Ciao3^−/−^ mice embryos was disordered, with the vascular lumen damaged and irregular. In wild‐type mouse embryos, the vascular network could be connected with each other after sprouting and growing, but in Ciao3^−/−^ embryos, the morphology of this network was not obvious, and the positive staining degree of CD31 in endothelial cells and endothelial progenitor cells was obviously reduced. The above results indicated that the vascular endothelial progenitor cells and endothelial progenitor cells of Ciao3^−/−^ embryo had maturation defects. Based on results from zebrafish and cell models, we speculate that the lethality of mouse embryos with Ciao3 deletion may also be due to increased oxidative stress and lipid peroxidation. Then, this speculation was verified, as the positive rates for 4‐HNE (Figure 6E) and BODIPY (Figure 6E) in Ciao3‐/‐ mouse embryos were significantly higher than those in wild‐type mouse embryos. At the same time, it was found by the γ‐H2AX method that the DNA damage of Ciao3^−/−^ mouse embryos was significantly enhanced (Figure 6H).

### 2.6 Deletion of Ciao3 Leads to Abnormal Expression of Ferroptosis Pathway‐Related Proteins in Mice Embryos

In an in vitro cell model, we found that lower Ciao3 expression could lead to the lower expression of GPX4, SLC7A11, and Ferritinm, but upregulation of TFR1 and IRP1. To verify the regulatory pathway in vivo, we conducted Western Blot experiments on 12.5‐day‐old wild‐type and Ciao3 knockout mouse embryos, and found that expressions of GPX4, xCT, and FTL were down‐regulated, whereas TFR1 and IRP1 were up‐regulated in Ciao3 knockout mice embryos. This up‐regulation of IRP1 will further lead to up‐regulation of TFR1 and lower expression of FTL, while lower expression of xCT and GPX4 will activate the ferroptosis pathway (Figure 6G,I). The above results are consistent with the cell model results.

### 2.7 Ciao3 Heterozygous Mice Develop Adult‐Onset Pulmonary Vascular Dysfunction

**Figure 7:**
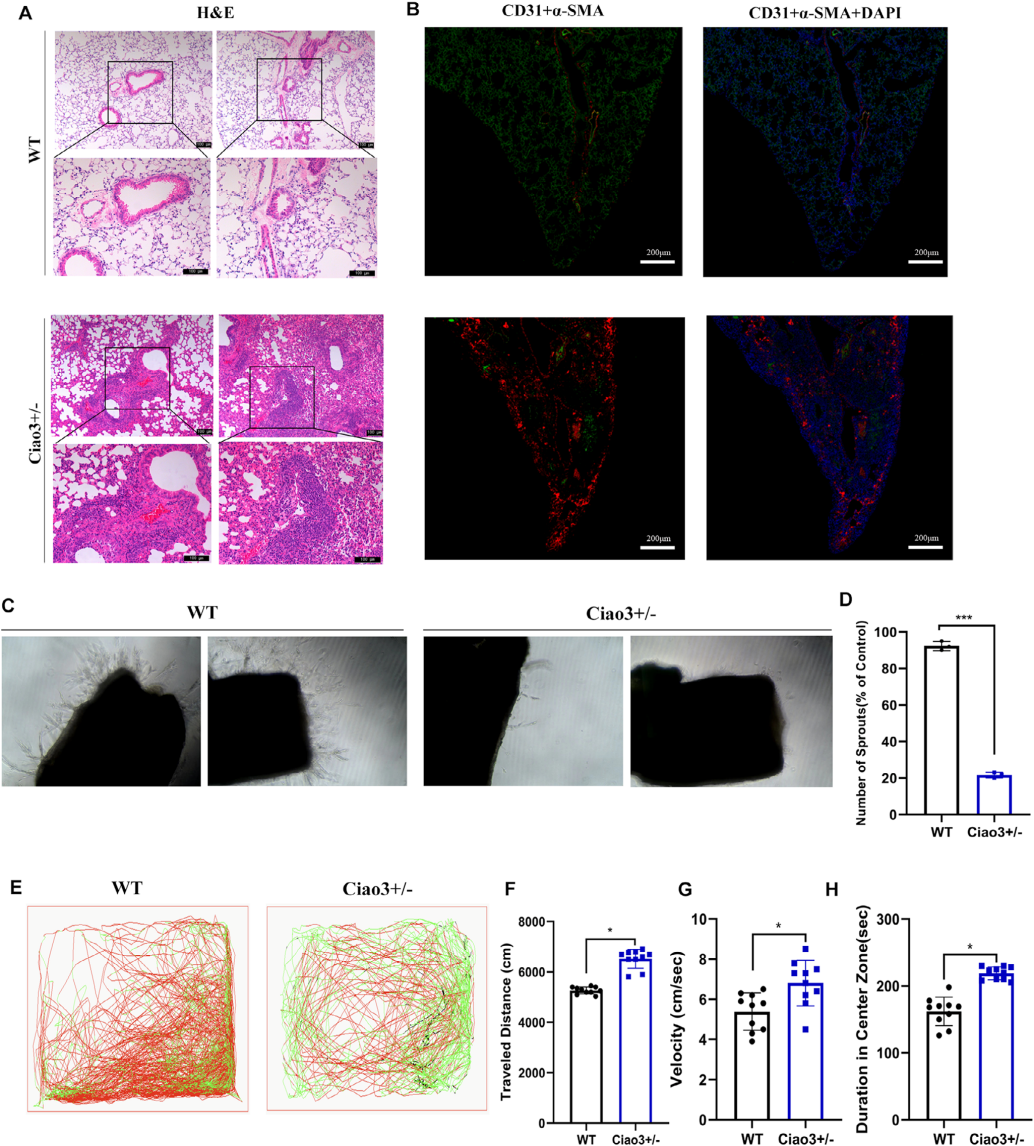
Impairment of Vascular Function in Ciao3 Heterozygous Mice. A) H&E staining results of lungs of 9‐week‐old mice; the lungs of Ciao3^+/−^ mice were significantly thicker than those of wild‐type mice in terms of pulmonary artery wall and small blood vessel ratio. B) CD31 and α‐SMA dual fluorescence immunostaining in lungs of 9‐week‐old mice; results showed that CD31 expression was evenly distributed across the lung lobes of wild‐type mice; CD31‐positive cells were densely distributed, whereas α‐SMA expression was rarely distributed. C,D) The aortic rings of mice in matrix glue were cultured in ECM medium for 4 days. The results showed that the number of buds in aortic rings from Ciao3^+/−^mice was significantly lower than in wild‐type mice. E, Illustrative example of mice movement in the open field test. F, Graphs showing the traveled distance, WT: 5268 ± 48 cm, n = 10; Ciao3^+/−^: 6518 ± 76 cm, n = 10. G, Graphs showing the velocity, WT: 5.4 ± 0.54 cm/s, n = 10; Ciao3^+/−^: 6.8 ± 0.63 cm/s, n = 10. H, Graphs showing the time in the center zone, WT: 162 ± 28s, n = 10; Ciao3^+/−^: 219 ± 16s, n = 10. Data are represented as mean±SEM. Values were obtained by unpaired Student's t‐test, not significant; *p < 0.05; **p < 0.01; ***p < 0.001.

Although there is little difference in adult phenotypes between Ciao3^±^ mice and wild‐type mice, it is observed that Ciao3^+/−^ mice are more fatigued compared with wild‐type mice from the 8th to 9th week. At the 9th week, the hearts, lungs, and livers of the two groups of mice were dissected and stained with HE. No difference was found between the heart and liver, but an obvious difference was found in pulmonary vessels (**Figure** **7**A). The results showed that compared to wild‐type mice, the pulmonary artery wall was significantly thicker, and the lungs had a significantly higher small blood vessel ratio in Ciao3± mice. Endothelial cell marker CD31 was shown to be evenly distributed in the lung lobes of wild‐type mice; CD31‐positive cells were densely expressed, and α‐SMA expression was rarely distributed (Figure 7B). However, CD31‐positive cells in the lung lobes of Ciao3^+/−^mice were significantly less than those of wild‐type mice, while α‐SMA expression was increased. The results indicated that the decrease of endothelial cells and the increase of smooth muscle cells in the lungs of Ciao3^+/−^mice, which might result in thickening of the pulmonary artery wall and stenosis of the pulmonary artery lumen. In order to detect whether the function of vascular endothelial cells in Ciao3^±^ mice was damaged, angiogenesis and vascular permeation experiments were carried out on the blood vessels of mice. The aortic rings of mice in matrix glue were cultured in ECM medium for 4 days. The results showed that the number of buds in aortic rings of Ciao3^+/−^ mice was significantly reduced compared with that of wild‐type mice (Figure 7C,D), suggesting that the angiogenesis of Ciao3^+/−^ mice was inhibited. We measured the vascular permeability of the aortic arch and the brain with albumin‐bound Evans Blue stain solution (Figure S6A, Supporting Information). The results showed that the Evans Blue of the aortic arch and cerebral vessels in Ciao3^+/−^ mice was significantly darker than in the wild‐type group, indicating increased vascular permeability in Ciao3^+/−^ mice. In addition, Ciao3‐deficient mice exhibited significantly exciting (13.5% ± 6.78%, p = 0.036; Figure 7E,F) and faster moving (12.8% ± 5.89%, *p* = 0.025; Figure 7G) than WT mice in the open field test. Moreover, they spent more time in the center of the open field (increased duration: 48% ± 19.8%; *p* = 0.042; Figure 7H). This might be a sign of epileptic seizures. All the above results indicated that the vascular function was impaired due to the Ciao3 deficiency.

### 2.8 *NARFL* Polymorphisms are Susceptible Sites of Vascular Endothelial Dysfunction Diseases

**Figure 8:**
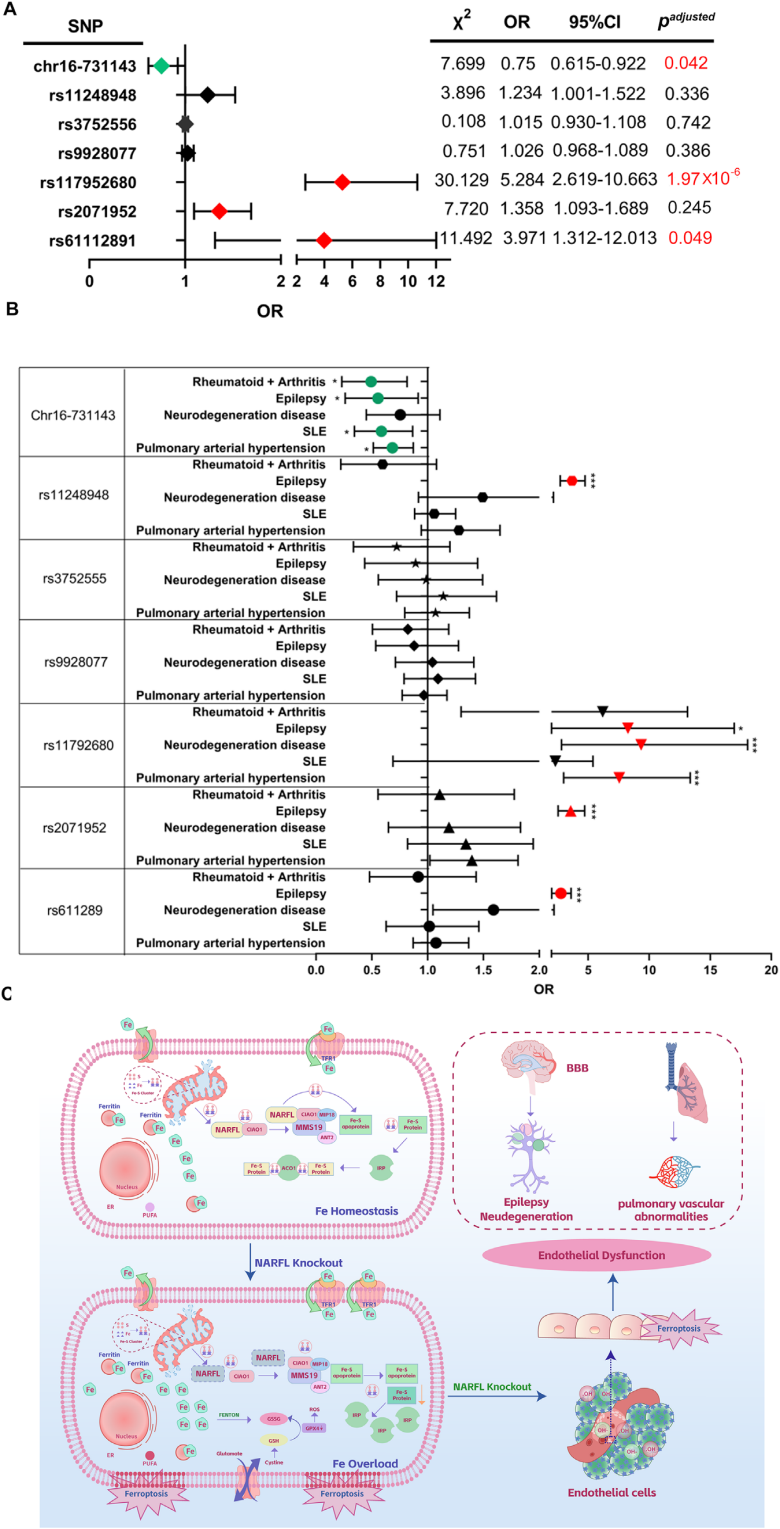
*NARFL* Polymorphisms are Susceptible Sites of Vascular Endothelial Dysfunction Diseases. A) Among the seven tagSNPs of *NARFL* rs61112891, rs2071952, rs117952680, rs9928077, rs3752556, rs11248948 and chr16‐731143, the frequency distribution of four SNP genotypes rs1179252680, rs2071952, rs61112891 and chr16‐731143, was statistically different between cases and controls (rs1179252680 (*p* < 0.001), rs61112891 (*p* < 0.01), rs2071952 (*p* < 0.05) and chr16‐731143 (*p* < 0.05)). The OR values of rs1179252680, rs2071952 and rs61112891 were greater than 1, while the OR value of chr16‐731143 was less than 1. B) The genotype frequency distribution was further analyzed between seven tagSNPs and patients with pulmonary hypertension, neurodegenerative diseases, epilepsy, systemic lupus erythematosus, rheumatoid and arteritis. C) Mechanism summary diagram: under normal circumstances, NARFL transfers the ISC synthesized in mitochondria to CTC composed of CIAO1, MIP18 and MMS19 through CIAO1, and imparts the ISC into specific apo proteins to form mature ferrithionein and maintain intracellular iron homeostasis. When NARFL is absent, NARFL cannot transfer the ISC synthesized by mitochondria to CTC, thereby preventing the formation of mature ferrithionein (ACO1). IRP1 increases the expression of TFR1 and inhibits the expression of Ferritin, resulting in increased iron intake. At this time, there is not enough ferritin to bind Fe and store it normally. As a result, intracellular iron ions increase and oxidative stress is enhanced via the Fenton reaction. At the same time, SCL7A11 and GPX4 are down‐regulated, and the synthesis of cytoplasmic glutathione and glutamine is reduced. This further increases the production of reactive oxygen species, activates lipid peroxidation, and induces iron death in vascular endothelial cells, leading to vascular endothelial dysfunction. Statistical significance was assessed using one‐way ANOVA with Tukey's multiple comparison correction. ns, not significant; *p < 0.05; **p < 0.01; ***p < 0.001.

We conducted a genetic association study comprising 387 patients with vascular endothelial dysfunction and 409 matched controls. The cohorts were well‐matched for age and sex distribution (case group: 50 ± 17.2 years, 55.8% male; control group: 48 ± 12.5 years, 55.7% male). Detailed demographic and clinical characteristics of the patient cohort are summarized in Table S1 (Supporting Information), which includes information on smoking/drinking status and specific disease subtypes: pulmonary hypertension with vascular involvement (48.3%), neurodegenerative diseases (13.2%), epilepsy (10.1%), systemic lupus erythematosus (17.1%), and rheumatoid arteritis (11.3%). Genotyping of seven NARFL tagSNPs identified four significantly associated with disease susceptibility (**Figure** **8**A; Table S2, Supporting Information). We found three risk genotypes: rs1179252680‐GA (OR = 5.284, 95% CI: 2.619‐10.663, *p* ^adjusted^ = 1.97 × 10^−^⁶), rs61112891‐GG (OR = 3.971, 95% CI: 1.312‐12.013, *p* ^adjusted^ = 0.049), and rs2071952‐CT/TT, while chr16‐731143‐TC/CC served as a protective genotype (OR = 0.75, 95% CI: 0.615‐0.922, *p* ^adjusted^ = 0.042). The remaining two SNPs showed no significant associations. Disease‐specific analysis revealed distinct genetic profiles (Figure 8B; Table S3, Supporting Information): rs11248948‐GG significantly increased cerebral small vessel epilepsy risk (OR = 3.588, 95% CI: 2.712‐4.746, *p* = 1.293 × 10^−1^ ^2^); rs117952680‐GA conferred elevated risks for cerebral small vessel epilepsy (OR = 5.826), neurodegeneration (OR = 7.129), and pulmonary hypertension (OR = 6.318); rs2071952‐TT/CT and rs611289‐GG/CG both increased cerebral small vessel epilepsy risk (OR = 3.462 and 2.699, respectively). The protective chr16‐731143‐TC/CC genotype reduced risks across multiple disease subtypes. It supports the genetic findings that the NARFL expression was significantly reduced in cases versus controls (Figure S6B, Supporting Information) and negatively correlated with the ferroptosis marker MDA (Figure S6C, Supporting Information). ROC analysis demonstrated that NARFL expression (AUC = 0.765) outperformed MDA levels (AUC = 0.540) in distinguishing risk genotypes (Figure S6D, Supporting Information), suggesting that NARFL under‐expression mediates the genetic susceptibility to vascular endothelial dysfunction.

## 3 Discussion

Our study establishes a critical and non‐redundant role for NARFL in maintaining vascular integrity. Using a multi‐model approach, we demonstrate that loss of NARFL or its orthologs consistently leads to severe vascular dysfunction and developmental defects. The embryonic lethality observed in both *narfl* ^−/−^ zebrafish and Ciao3^−/−^ mice underscores the fundamental requirement of NARFL for survival, primarily attributed to failed vascular development. In zebrafish, this manifests as specific and profound structural malformations of major vessels, including the dorsal aorta (DA) and posterior cardinal vein (PCV), accompanied by aberrant hemodynamics and a loss of endothelial functional homeostasis, as indicated by dysregulated ET‐1 and NO. The recapitulation of key phenotypes in NARFL^−/−^ HPMEC, including impaired proliferation, tube formation, and barrier function, confirms that the endothelial cell‐autonomous defects are a primary driver of the systemic pathology.

The convergence of phenotypic evidence across models led us to investigate the underlying molecular pathway. We identify a coherent mechanistic cascade originating from the disruption of the cytosolic iron‐sulfur cluster assembly (CIA) machinery. As a core component of the CIA system, NARFL deficiency disrupts the maturation of cytosolic Fe‐S proteins. A pivotal consequence is the functional switch of the bifunctional protein ACO/IRP1. The loss of its Fe‐S cluster converts ACO, which has a metabolic role, into the RNA‐binding protein IRP1. This shift unlocks a pathological sequence: hyperactive IRP1 binds to iron‐responsive elements (IREs), leading to the sustained upregulation of the transferrin receptor (TFR1) and simultaneous suppression of ferritin expression. This disrupted iron homeostasis results in catastrophic intracellular iron overload. The iron overload, in turn, acts as a potent catalyst for the Fenton reaction, generating massive amounts of reactive oxygen species (ROS) and inducing extensive lipid peroxidation. Concurrently, we observed downregulation of key defensive pathways, including the cystine/glutamate antiporter (SLC7A11/xCT), glutathione peroxidase 4 (GPX4), and the protective cytochrome P450 epoxygenase CYP2J2. The combination of unchecked lipid peroxidation and crippled antioxidant defenses creates a perfect storm that drives endothelial cells into ferroptosis, a non‐apoptotic, iron‐dependent form of cell death. This is further corroborated by the characteristic mitochondrial morphology and the significant rescue of viability and function by the ferroptosis inhibitor, Ferrostatin‐1, across our models.

Our findings extend the pathological implications beyond developmental vasculature to the mature neurovascular unit. Using a Narfl knockout zebrafish model combined with the Tg (*flk*:eGFP) transgenic line to visualize vascular dynamic,^[^ ^22^ ^]^ we demonstrated that Narfl deficiency causes juvenile lethality, epilepsy‐like behavior, BBB disruption, and neuronal pathology. These findings parallel clinical observations of *NARFL* mutations in an epileptic family,^[^ ^23^ ^]^ supporting a conserved role for NARFL in neurovascular homeostasis. While TEM revealed ultrastructural BBB defects in mutants (e.g., endothelial tight junction abnormalities), we acknowledge that tracer‐based functional assays (e.g., 1 kDa NHS, 10 kDa dextran, or 80 kDa DBP‐EGFP permeability tests) could provide more dynamic and quantitative insights into BBB integrity. Future studies employing these tracers will clarify whether NARFL loss selectively compromises size‐dependent vascular permeability. The pathogenesis of epilepsy is complex, and one of the mechanisms is the imbalance of central nervous system homeostasis caused by BBB injury.^[^ ^15^, ^24^ ^]^ In addition, we also found that Narfl deletion led to abnormal vascular development and structure in zebrafish. In previous studies, we have found that knocking out Narfl led to abnormal intestinal vessels in zebrafish. We observed the whole process of vascular development of zebrafish more intuitively through Tg (*flk*: eGFP) model, and found that Narfl deletion led to deformity or even absence of dorsal longitudinal anastomosis vessels (DLAV) and connecting cells in the development process of zebrafish, as well as distortion of DA and PCV. In follow‐up experiments, we found that NARFL deletion led to increased oxidative stress, lipid peroxidation, and iron levels. It is speculated that increased lipid peroxidation, driven by elevated free iron and oxidative stress, will injure vascular endothelial cells in zebrafish, leading to blood vessel malformations during development. When measuring the blood flow of zebrafish, we found that the blood flow of *narfl* ^−/−^ zebrafish increased significantly before 6 dpf, reached its peak at 6 dpf, and decreased significantly after 6 dpf. However, the blood flow velocity of wild zebrafish did not change significantly. To investigate the reasons, we examined the endothelial markers ET‐1 and NO. We found that ET‐1, responsible for vasoconstriction, increased obviously at 6 dpf, while NO, responsible for vasorelaxation, decreased obviously at 6 dpf, but this phenomenon did not occur at 4 dpf. We speculated that endothelial cells still compensated after injury before 6 dpf, but the relaxation and contraction functions of endothelial cells were obviously damaged at 6 dpf, and almost all *narfl* ^−/−^ died before 13 dpf, which indicated that Narfl deletion would lead to serious damage to the function of vascular endothelial cells. We observed the vascular development of zebrafish and found that the DA and posterior aorta of *narfl* ^−/−^ zebrafish did not fuse to form a regular circulation as in wild‐type zebrafish, but formed a distorted and disorganized shape with each other. The formation of DA and PCV occurs during the early stage of embryonic angiogenesis. When zebrafish develop to ≈15 nodules, angioblasts in the middle layer of the lateral plate converge at the midline to form DA and PCV.^[^ ^25^ ^]^ Since angiogenesis involves the remodeling and maturation of the vascular network, a precursor of endothelial cells can only differentiate into an artery or a vein, whereas *narfl* ^−/−^ zebrafish exhibit abnormalities in the processes of angiogenesis, generation, and differentiation.

Transcriptomic and functional analyses revealed that Narfl knockout significantly reduced *cyp2p8*(human CYP2J2) expression in zebrafish vasculature. As a cytochrome P450 epoxygenase, CYP2J2 metabolizes arachidonic acid to anti‐inflammatory epoxyeicosatrienoic acids (EETs) and mitigates oxidative damage by converting H_2_O_2_ into hydroxyepoxy metabolites.^[^ ^26^, ^27^ ^]^ Cyp2p8 uses molecular oxygen to insert an oxygen atom into the substrate and reduce the second oxygen atom into water molecules. CYP2J2 is widely expressed in vascular endothelial cells.^[^ ^28^ ^]^ CYP2J2 and its products have protective effects on vascular injury. They can convert hydrogen peroxide into hydroxyepoxy metabolites, participate in eicosanoic acid metabolism, combine with 15‐lipoxygenase to metabolize arachidonic acid, and convert hydroperoxicosatetraenoates (HpETEs) into hydroxy epoxy eicosatrienoates (HEETs).^[^ ^29^ ^]^ HEETs play a protective role in vascular injury through various mechanisms such as anti‐inflammation, anti‐apoptosis, and inhibition of vascular endothelial cell aging.^[^ ^29^, ^30^ ^]^ It can be concluded that when the expression of CYP2J2 decreases, both HEETs and the protective effect of blood vessels reduce. Through qRT‐PCR and whole‐mount in situ hybridization (WISH) experiments, we found that cyp2p8 (CYP2J2 in humans) expression in *narfl* ^−/−^ zebrafish is significantly decreased compared to wild‐type zebrafish.

The preface states that deleting NARFL would hinder ISC transmission to IRP1, and that ACO would lose ISC and become IRP1, leading to a decrease in ACO activity. It has been reported that,^[^ ^31^, ^32^, ^33^, ^34^ ^]^ whether in lower yeast or higher mammalian cells, the deletion of mitochondrial ISC synthesis‐related proteins has the following similar phenotypes: excessive iron in mitochondria leads to increased oxidative stress, blocked electron transmission, decreased mitochondrial function, and the decrease of iron in cytoplasm will up‐regulate IRP1 activity, resulting in an increase in iron uptake. We found that NARFL deletion did not affect mitochondrial cis‐aconitase activity but decreased cytoplasmic cis‐aconitase activity, leading to iron overload; therefore, we wanted to determine whether mitochondrial function is affected by NARFL. Using the Seahorse XFe analyzer, we found that NARFL deletion decreased mitochondrial respiratory function. We hypothesized that NARFL deletion might increase IRP1 activity, induce high TFR expression, and increase intracellular iron intake, leading to an inability to effectively utilize mitochondrial iron, aggravating mitochondrial iron overload, increasing oxidative stress, and hindering electron transfer. However, whether this mechanism holds requires further cell experiments.

Corbin et al. found that *NARFL* gene overexpression existed in two oxygen‐resistant strains of HeLa cells through genome‐wide DNA and RNA array analysis combined with functional genomics research; They also found that hyperoxia‐induced overexpression of NARFL can protect the activity of iron‐sulfur proteins ACO, thus revealing the key role of NARFL in resisting oxidative stress induced by hyperoxia.^[^ ^35^, ^36^, ^37^ ^]^ Like the results of knocking out NARFL homologue Nar1 in yeast, knocking out NARFL will lead to cytoplasmic iron‐sulfur protein assembly defect and body and cell death, which confirms the important role of NARFL in the cytoplasmic iron‐sulfur protein assembly pathway.^[^ ^38^ ^]^ Fan XR et al.^[^ ^38^ ^]^ found that the *NARFL*‐S161I mutant failed to bind the functional CIA complex, and we speculated that this mechanism was related to diffuse pulmonary arteriovenous malformation caused by this mutation, which we first discovered. Their study also found that the association between NARFL and CIA complex was closely related to cellular iron levels. The binding of NARFL and CIA complex was also affected by oxidative stress level and hypoxia. The interaction between NARFL and CIA complex was enhanced when iron supplementation or hypoxic tension was added, while reactive oxygen species weakened the interaction between NARFL and CIA components.

According to the results of zebrafish and cell model experiments, the conclusion can be put forward: under normal circumstances, NARFL transmits ISC synthesized in mitochondria to CTC, composed of CIAO1, MIP18, and MMS19 through CIAO1, and inserts ISC into specific apoproteins to form mature iron‐sulfur proteins and maintain iron homeostasis at the same time. However, when NARFL is absent, it cannot transmit ISC synthesized in mitochondria to CTC, which leads to an inability to form mature iron‐sulfur proteins. For example, when cytoplasmic ACO cannot obtain ISC, it acts as an “RNA binding protein” IRP1. IRP1 increased the expression of TFR1 and inhibited the expression of Ferritin, which led to an increase in iron intake. There was not enough Ferritin to bind and store Fe normally, leading to increased intracellular iron ions and further oxidative stress via the Fenton reaction. SCL7A11, GPX4, and CYP2J2 were down‐regulated. The decrease in glutathione and glutamine synthesis in the cytoplasm further increases reactive oxygen species production and activates lipid peroxidation, leading to ferroptosis and vascular endothelial cell death and dysfunction. ACO is the link between iron metabolism balance and the oxidative stress signal pathway. The decrease of ACO activity in the cytoplasm and the increase of free iron contents would promote the production of ROS. IRP1 functions as an iron receptor in the cytoplasm. Normally, when the iron content of cells increases, IRP1 binds with [4Fe‐4S] and converts into ACO. When the iron content of cells decreases, IRP1 dissociates from [4Fe‐4S] and binds with IREs in the non‐coding region of iron metabolism‐related protein mRNA, which promotes iron absorption and restores iron levels but reduces free iron storage in cells. For example, there are five IRE structures in the 3′UTR of TFR mRNA. When cells are deficient in iron, IRP can bind to IRE in TFR genes to protect their mRNA from ribonuclease degradation, resulting in increased TFR levels and iron absorption in cells.^[^ ^39^ ^]^ IRE of Ferritin exists in 5′ UTR, and when cells are deficient in iron, IRP1 combines with IRE of Ferritin 5 ′UTR, which reduces Ferritin synthesis, resulting in reduced combined iron and utilization in cells. NARFL plays a very important role in this balance process. Once NARFL is deleted, this balance is broken.

Song et al. is the only team that has completed the research report of the mouse *Ciao3* gene knockout. The team found that mice died 10.5 days in embryo after *NARFL* gene knockout, and induced acute Ciao3 overall knockout in adult mice will lead to the death of adult mice, and the cytoplasmic aconitase activity in their liver decreases significantly; Knockout of Ciao3 in mice embryonic fibroblasts will lead to the decrease of cytoplasmic aconitase activity and cell viability.^[^ ^10^ ^]^ We found that Ciao3^−/−^mice died or were absorbed in the early embryonic stage (E 12.5 and before), which is consistent with Song's conclusion that Ciao3^−/−^mice embryos died or were absorbed before 10.5 days. One of the earliest events in embryonic development is the formation of blood vessels, and mesodermal cells differentiate into vascular cells, namely hematopoietic progenitor cells and endothelial progenitor cells, beginning on the 7th day. Thus, we speculate that Ciao3 deletion would damage endothelial progenitor cells. It was found that Ciao3‐/‐ mouse embryonic endothelial progenitor cells had maturation defects, leading to failure to connect endothelial cells into a vascular network in the early embryo. It was reported that vascular endothelial progenitor cells form functional circulation in the early stage;^[^ ^40^ ^]^ Vascular progenitor cells would respond to basic fibroblast growth factor and bone morphogenetic protein 4 in the posterior primitive stripe in the form of vascular endothelial growth receptor‐2 (flk1) positive mesodermal cells; flk1 positive cells in the primitive stripe produce blood and endothelial cells at the same time, but they are limited to hematopoiesis or angiogenesis after migrating to the outer and inner embryos.^[^ ^41^ ^]^ In the yolk sac, these endothelial progenitor cells aggregate into endothelial‐lined blood islands and then fuse to form the primary capillary plexus, which undergoes remodeling together with intracellular blood vessels to form mature circulation. If the maturation cycle of endothelial progenitor cell formation is not established, the embryo would stop developing and die.^[^ ^42^, ^43^ ^]^ We hypothesize that BBB leakage in Ciao3 KO embryos, potentially exacerbated by tracer detectable paracellular flux, may synergize with circulatory collapse to deprive developing tissues of oxygen/nutrients, accelerating embryonic resorption.

Compared with wild‐type embryos, we also observed a significant increase in oxidative stress, lipid peroxidation, and DNA double‐strand breaks in Ciao3‐/‐ mouse embryos. During embryonic development, excessive ROS can break deoxyribonucleic acid, peroxidize proteins and lipids, and finally lead to mitochondrial damage. Embryonic mitochondrial DNA defects can induce metabolic dysfunction, which leads to embryo damage, embryo development retardation, and even stagnation.^[^ ^44^ ^]^ The production of ROS is affected by many factors, and the amount produced varies across different stages of embryo development. For mouse embryos, ROS is produced mostly in fertilization and G2/M stage of the second cell stage, and iron ions can directly act on lipids and amplify peroxidation damage caused by free hydroxyl radicals.^[^ ^45^ ^]^ Lipids are important substances that constitute the cytoskeleton. Lipid peroxide is formed after the combination reaction of PUFA and oxygen‐free radicals in vivo. When oxidative stress is excessive, lipid peroxide production increases, which can dissolve PUFA in the cell membrane, disrupt its structure, alter its fluidity and permeability, and then affect cell metabolite transfer and cell signal transduction.^[^ ^46^ ^]^ Embryonic development stagnation is also a self‐protection mechanism during embryogenesis, aimed at preventing abnormal or low‐quality embryos from progressing further. Western Blot experiment showed that GPX4, xCT, and FTL expressions were down‐regulated in Ciao3^−/−^mice embryos. The expression of TFR1 and IRP1 was up‐regulated, which was consistent with the results of the cell model. Therefore, we infer that NARFL deletion can lead to up‐regulation of IRP1 expression in the mouse model, which in turn leads to up‐regulation of TFR1 and lower expression of FTL. Ciao3 deletion also induces lower expression of xCT and GPX4, thus activating the ferroptosis pathway. This increased oxidative stress and lipid peroxidation in Ciao3^−/−^embryo endothelial progenitor cells, resulting in the obstruction of endothelial progenitor cell maturation and circulation, and ultimately leading to early embryo death. We didn't find much difference between Ciao3±mice and wild‐type mice, but we found that there was obvious endothelial cell injury in the lungs of Ciao3^+/−^mice in the later stage. There was an obvious thickening of the wall of pulmonary blood vessels, a decrease in endothelial cells, and an increase in smooth muscle. In the mouse model, we also found that the absence of Ciao3 impaired the maturation and differentiation of endothelial progenitor cells. Endothelial progenitor cells differentiate into veins and arteries and gather in primitive capillaries. Neovascularization is initially composed of endothelial cells. Vascular maturation requires the integration of vascular and arterial factors for a sufficient period, so that endothelial cells can be tightened and covered by parietal cells and extracellular matrix. If endothelial cells are damaged, blood vessels will leak, be fragile, easily ruptured, and bleed, which will also reduce blood vessel flow and lead to vascular degeneration.^[^ ^47^, ^48^, ^49^ ^]^

Gene Curation^[^ ^50^ ^]^ is extracted from the literature and databases based on the current research status of the gene's evidence to assess the strength of clinical validity of the “gene‐disease” relationship. NARFL was first reported in relation to pulmonary hypertension secondary to diffuse pulmonary arteriovenous malformation. According to the ClinGen Gene Curation Standardized Evidence SOP version 8, this case can score 0.5 points in Case‐Level Data evidence and 6 points in Case‐Control Data evidence. This suggests that NARFL may be associated with pulmonary hypertension. The study of NARFL originated from a rare family with pulmonary arterial hypertension secondary to a diffuse pulmonary arteriovenous malformation, in which a missense mutation in NARFL led to severe consequences. In a genetic study, we typically identify the pathogenic mutation in a gene based on a family's phenotype, and then explore the gene's pathogenic mechanism using this clue. The study of rare diseases and their pathogenesis is both challenging and meaningful. However, by observing the phenotype of *Ciao3^+/^* ^−^mice at a later stage, we wonder whether there are susceptible sites in the NARFL gene that cause vascular endothelial dysfunction. Furthermore, we'd like to bridge the mechanisms of rare diseases to those of common disease susceptibility. The identification of specific NARFL tagSNPs (rs11248948, rs117952680) associated with an increased risk of pulmonary hypertension, epilepsy, and neurodegenerative diseases in our clinical cohort suggests a spectrum of NARFL‐related endothelialopathies. The significant reduction in NARFL expression in cases. And its superior predictive value over MDA levels strengthens the hypothesis that even a partial reduction in NARFL function, potentially mediated by these non‐coding variants, can predispose individuals to vascular endothelial dysfunction.

In conclusion, our data unveil a previously unrecognized pathway in vascular biology: NARFL safeguards endothelial health by ensuring cytosolic Fe‐S protein maturation, thereby preventing IRP1‐mediated iron overload and subsequent ferroptosis. The disruption of this pathway, whether by complete knockout or hypomorphic polymorphisms, leads to a continuum of diseases, from embryonic lethality to adult‐onset pulmonary and neurovascular disorders. This work not only elucidates the pathogenesis behind a rare vascular malformation but also positions NARFL and the CIA‐ferroptosis axis as a compelling frontier for understanding and treating a wider range of vascular endothelial dysfunction diseases.

## 4 Conclusion

In summary, our multi‐model investigation demonstrates that NARFL is indispensable for vascular endothelial integrity by playing a central role in the cytosolic iron‐sulfur cluster assembly pathway. Disruption of NARFL function impairs Fe‐S protein maturation, leading to aberrant ACO/IRP1 switching, iron overload, and oxidative stress, ultimately driving endothelial ferroptosis. This mechanism underlies the embryonic lethality observed in *narfl* ^−^ *^/^* ^−^ zebrafish and Ciao3^−/−^ mice, and is further implicated in adult‐onset vascular pathologies via NARFL polymorphisms associated with pulmonary hypertension, epilepsy, and neurodegenerative diseases. Our findings not only elucidate a previously unrecognized ferroptosis‐driven pathway underlying vascular dysfunction but also highlight NARFL as a potential therapeutic target and genetic susceptibility factor in a spectrum of endothelial disorders.

## 5 Experimental Section

### Generation of Narfl Mutant Lines and RNA Injection

Narfl knockout was performed using a CRISPR/Cas9 system as previously described.^[^ ^17^ ^]^ Homozygous mutants were obtained from F1 embryos derived from F0 and wild‐type intercross. Two mutant lines, namely *narfl^−/−^*‐1 (harboring a 4 bp insertion) and *narfl^−/−^*‐2 (harboring a 5 bp deletion plus a 4 bp insertion), were genotype‐identified using primers: F‐5′‐TAATACGACTCACTATAGATGTTGGCGTCTGCAT GTCCGTTTTAGAGCTAGAAATAGC‐3′; R‐5′‐AGCACCGACTCGGTGCCACT‐3′. For narfl mRNA injection, the full length of zebrafish narfl cDNA was cloned into pSP64‐poly A vector and synthesized into the capped mRNA using an mME SSAGE mMACHINE SP6 Kit (Ambion, Austin, TX, USA). About 300 ng µL^−1^ of narfl mRNA was injected into 1‐2‐cell‐stage embryos.

### Zebrafish and Behavioral Analysis

The culture of zebrafish and all experimental procedures were conducted according to the zebrafish handbook and with the regulations of the Care and Use of Laboratory Animals under the approval of the Institute of Hydrobiology, Chinese Academy of Sciences (Approval ID: IHB 2013724). The wild‐type zebrafish was maintained in our laboratory under standard conditions.^[^ ^17^ ^]^ Zebrafish embryos' developmental stages were determined by hour post‐fertilization (hpf) or dpf.^[^ ^51^ ^]^ The *flk: GFP/narfl* ^−/−^ homozygous lines were generated by mating the *flk: GFP/narfl^+/^* ^−^ *a*dult fish, which were obtained by hybridizing the *flk: GFP/Con* and *narfl^+/^* ^−^ fish. 7 dpf zebrafish were placed in the 24‐hole plate with one zebrafish in each hole. The 24‐hole plate was placed in the behavior analysis system using Viewpoint zebrafish tracking software (ViewPoint Life Sciences, Zebraoo1, Lyon, France) for analysis, the detection area was set, and the parameters were adjusted: Time (60 min), background pixels (18‐24 pixels), speed (0.4‐10 mm/s), output interval time (60 s), photcycle intensity (500 lx). To ensure sufficient data could be obtained from zebrafish of different genotypes, 10 experimental groups were established. A total of 240 zebrafish behavior datasets were monitored and their track maps were generated. View Point's Micro Zebra Lab uses high‐speed video recordings of zebrafish to capture data on pulse rate, blood flow, and changes in vessel diameter. The software algorithm calculates the blood flow data by analyzing the correlation between the two frames. Take the zebrafish with 3–13 dpf and place it on the slide. Open the video file on the computer, place the slide with the zebrafish sample on the microscope, select the observation area in the box, and analyze for 1 min, making sure the settings were correct. The red represents the original measured data, and the blue represents the results after filtering by the fast Fourier Transform algorithm (FFT).

### Blood Vessels and Blood‐Brain Barrier in Zebrafish and Imaging

Confocal images of 3dpf *flk: GFP/narfl* ^−^ *^/^* ^−^ homozygous embryos were generated using a Zeiss ISM 710 confocal microscope, to observe Blood‐brain barrier (BBB), Dorsal longitudinal aorta vessels (DLAV), Dorsal aorta (DA), Posterior cardinal vein (PCV), connector cells, and base cells. Calculate the percentage of total DLAV, junction cells, and basal cells performed as previously described.^[^ ^52^ ^]^ The ultrastructure of the BBB in zebrafish was observed by transmission electron microscopy. 7 dpf zebrafish with different genotypes were fixed, dehydrated, transparent, waxed, embedded, sliced (thickness of 6 µm), stained with toluidine blue, and Nissl bodies were observed under a microscope. At the same time, hydrochloric acid and potassium ferricyanide solution were mixed in a 1:1 ratio to prepare Prussian blue stain, which was then applied as described above to observe the positive situation.

### Whole‐Mount RNA in situ Hybridization (WISH) and Quantitative Real‐Time PCR

*cyp2p8* was synthesized, Forward primer: 5′‐AGGAAACATCCGTC ATGGACT‐3′ and Reverse primer: 5′‐TAATACGACTCACTATAGGG (T7) ATGGCTTAGGACAGTGTGTGC‐3′.WISH was performed as previously described.^[^ ^53^, ^54^, ^55^ ^]^ Total RNA was extracted from the embryos at different developmental stages using the Trizol reagent (Invitrogen Carlsbad, CA, USA) and quantitative real‐time PCR was performed as previously described.^[^ ^17^ ^]^ Data were analyzed using a ^△△^Ct method and *β*‐actin served as the housekeeping gene. All the experiments were performed in triplicate and the primers are listed in Table S4 (Supporting Information).

### Construction of a Stable Cell Line with NARFL Gene Knockout in HPMEC Cells

The *NARFL* gene has two protein‐encoding genes. For expression sequences, the experiment synthesized data from NCBI and Ensemble to encode 476 aa and 374 AA selections, the common exon region of aa transcripts was selected to design screening SgRNAs. The sgRNA location and the sequence are shown in Figure S4A (Supporting Information). Wild‐type HPMEC cells in normal culture were collected and tested using the TIANamp Genomic DNA Kit. Plasmid DNA was extracted from the reagent box, and PCR amplification was performed NARFL target gene with 2×EasyTaq PCR SuperMix. Five SgRNAs were confirmed for the construction of the sgRNA‐Cas9 plasmid. HEK293 cells were inoculated the day before virus packaging, and the virus density exceeded 80% the next day. The mixture was prepared and packaged according to the lentivirus system, and added to the cultured HEK293T cells, and cultured for 96 h. After 96 h, the virus was collected and filtered for cell infection. After lentivirus packaging was completed, each Cas9‐sgRNA venom was infected into HPMEC cells under the same conditions as the control GFP venom. One week after drug screening, some cells were collected for genomic PCR amplification to determine whether the corresponding sgRNA had cleavage activity. The remaining verified cells were inoculated into a 96‐well plate with one cell per well, and cultured for two weeks. The monoclonal cell community in the 96‐well plate was selected and expanded into the 24‐well plate, and then expanded into the 6‐well plate after overgrowing. Genomic DNA amplification targets were extracted from expanded monoclonal cell lines and sequenced to determine whether the target genes were successfully edited and to obtain the target gene knockout cells (Figure S4B, Supporting Information). A total of 109 monoclonal cells were selected in the process of monoclonal cell selection, but only 4 monoclonal cells (HPMEC‐*NARFL*‐sg6‐10, 20, 26, 40) detected functional knockout of single alleles. However, during the culture of these four monoclonal cells, it was found that HPMEC‐*NARFL*‐sg6‐10 died during growth and could not meet the experimental requirements. Therefore, HPMEC‐*NARFL*‐sg6‐20, 26, and 40 were selected for further verification by Western blot assay (Figure S4C, Supporting Information). The results of the western blot showed that the expression of HMEC‐*NARFL*‐sg6‐40 decreased most significantly compared with wild‐type cells, and HPMEC‐*NARFL*‐sg6‐40 was finally selected for follow‐up experiments (Figure S4D, Supporting Information).

### Ferroptosis‐Related Indicators Assay

Caudal fins of zebrafish embryos were cut for genomic DNA isolation and genotyping using the NaOH lysis method as previously described^57^. After the fish tail genotyping, the rest of the body was sampled for the ROS assay. Briefly, the embryo was digested with 100 µL of 0.25% (w/v) trypsin/EDTA solution for 10 min, after which 200 µL of DMEM containing 10% (v/v) fetal bovine serum (FBS) was added to stop the reaction. The sample was centrifuged at 2500 rpm for 5 min at 4 °C to remove the supernatant, and the cell pellet was washed in 200 µL of PBS containing 2% (v/v) FBS. After a second centrifugation, cells were resuspended in 200 µL of PBS containing 2% FBS with 10 µM DCFH‐DA probe, BODIPY 493/503 probe, and DPPP probe (Maokang, Shanghai, China), and incubated at 37 °C for 30 min. A sample without probe incubation served as a negative control. Fluorescence was detected at the excitation/emission wavelength of 488/525 nm and 351/380 nm using a FACS Canto Flow Cytometer (BD Bioscience, USA). 5 dpf zebrafish embryos cultured at 1×PTU were collected and put into a 24‐well cell culture plate and replaced with new egg water. 1 µL BES‐H2O2‐AC fluorescent dye (1 mg/L, soluble in DMSO), BODIPY 493/503 and DPPP were added to each well at a dilution of 1:10000. The dye solution was quickly dispersed by shaking gently. The incubator was placed in a 28 °C incubator for 2 h, then removed for observation under a fluorescence microscope. To inhibit ferroptosis, 16 µM Ferrostatin‐1 (MedChemExpress, LLC, USA) was used for exposure starting at 24 hpf; 3 dpf embryos were collected for ferroptosis measurement after two days of treatment.

### Cell Counting Kit‐8 (CCK‐8) Cell Proliferation Experiments

CCK‐8 uses water‐soluble tetrazolium salt‐WST‐8 (2‐ (2‐methoxy‐4‐nitrophenyl) ‐3‐ (4‐nitrophenyl) ‐5‐ (2, 4‐disulfonyl benzene) ‐2h‐tetrazolium monosodium salt developed by Dojindo. It can be reduced to water‐soluble methyl dyes in the presence of the electron carrier 1‐Methoxy PMS. The orange‐yellow formazan dye produced by 2‐ (2‐methoxy‐4‐nitrophenyl) ‐3‐ (4‐nitrophenyl) ‐5‐ (2, 4‐disulfonobenzene) ‐2h‐tetrazolium monosodium salts, redoxed by intracellular dehydrogenase, can be dissolved in the tissue medium, and the amount of formazan produced was proportional to the number of living cells.

### Biochemical Analyses

Cytosolic aconitase activity was determined using an Aconitase Activity Assay Kit (Sigma‐Aldrich, St. Louis, MO, USA). Cellular redox substances, including glutathione (GSH), glutamine, and malondialdehyde (MDA), were measured by colorimetry assay using commercial kits (Beyotime, Nanjing, China). The iron level of zebrafish was determined by colorimetry using a commercial assay kit (Dojindo Laboratories, Kumamoto, Japan). Vascular endothelial function, including Nitric oxide (NO) and Endothelial 1 (ET‐1), was detected by ELISA using commercially available assay kits (LMAI, Shanghai, China).

### Seahorse Assay

In 5dpf zebrafish and HPMEC (20000 cells/well), oxygen consumption rate and extracellular acidification rate (a surrogate marker of glycolysis) were measured on an XFe24 Extracellular Flux Analyzer (Seahorse Biosciences) by sequential addition of 1 µM Oligomycin, 0.5 µM FCCP, and 2 µM Rotenone plus 0.5 µM Antimycin, as previously described.^[^ ^56^ ^]^

### In Vitro Angiogenesis Assays

Tube formation was performed using the in vitro angiogenesis assay kit (Chemicon International, Temecula, USA). Briefly, Matrigel with reduced growth factors was pipetted into pre‐chilled 48‐well plate (100 µl Matrigel per well) and polymerized for 30 min at 37 °C. HPMECs and HPMEC‐NARFL^−^ *^/^* ^−^ with different treatments (2◊10^5^ cells per well) resuspended in 100 µl of basic media, and seeded in a Matrigel‐coated 48‐well plate. After 4–6 h of incubation, tubular structures were photographed using an Olympus microscope with 20◊magnification. After tube formation, photos were taken, and the angiogenesis analysis plug‐in in Image J was used to analyze nodes, intersections, mesh number, mesh area, vascular branch number, total vascular length, vascular branch length, and trunk length in the acquired images. The number of branch points was quantified in triplicate determinations from 3 separate experiments.

### A Ciao3^+/−^ Mouse Model was Generated using the CRISPR/Cas9 Method

The Ciao3^−/−^ hybrid was constructed in C57BL/6 mice by CRISPR/Cas9. The Ciao3 motif (NCBI: NM_026233.8; Chromosome 17 Ensembl: ENSMUSG000 00002280) in mice, a total of 11 exons (transcript Ciao3‐201: ENSMUST0 0000002350), exon 3–4 as the knockout target, exon 3 starts from ≈11.41% of the coding region, exon 3–4 accounts for 19.4% of the coding region, and the effective knockout region size was 1820 bp. The Cas9 and gRNA combination was injected into a fertilized egg, and the offspring were targeted for knockout. F0 was identified by PCR, and wild‐type mice were propagated to detect germ‐line transmission and F1 offspring, and heterozygous mice were hybridized to produce homozygous generations. Gene identification strategy: Primer1:F1:5′‐CTGGCTCAGACCATTTCTGCATC‐3′; R1: 5′‐GTGATGCTGCCAAACACTC GTCA‐3′. Wild‐type fragment size: 2509 bp; The mutant fragment size was 683 bp. Primer 2: F1:5′‐CTGGCTCAGACCATTTCTGCATC‐3′; R1:5′‐ TTTTCTATT TCCTGACAGTAGGTGG‐3′. Wild type fragment size: 523 bp. The heterozygous fragments are as follows: the fragment size of primer 1 was 683 bp, and the fragment size of primer 2 was 523 bp.

### Immunoblotting and Co‐Immunoprecipitation

Cells were lysed in Laemmli buffer, protein lysates were resolved by SDS‐PAGE and transferred onto a PVDF membrane. Membranes were blocked in 5% non‐fat milk or BSA in PBS containing 0.1% Tween (PBST) and incubated overnight at 4 °C with the primary antibody, followed by incubation with secondary antibodies for 1 h at room temperature. After washing in PBST buffer, immunoreactive bands were visualized with the ECL system. Primary antibodies for NARFL (NOVUS, 1/1000) and GPX4 (NOVUS, 1/1000) were obtained from NOVUS, TFR (PK17158, 1/500), Ferritin (T55648, 1/1000) and IRP1 (T55075, 1/1000) were obtained from Abmart, SLC7A11 (A2413, 1/2000) was obtained from CST, CYP2J2 (ATA27790, 1/2000) was obtained from Atagenix, CIAO3 (sc‐514078, 1/500), MMS19 (sc‐390028, 1/500), FAM96B (sc‐376801, 1/5000), MMS19 (sc‐390028), and CIAO1 (sc‐374498, 1/500) were obtained from santa, GAPDH (ab8245, 1/6000) was obtained from Abcam. Mouse embryos were cut into fine fragments, the lysate was added at a ratio of 200 µL per 20 mg of tissue, homogenized until complete, centrifuged at 4 °C 12 000 g for 15 min, and the supernatant was taken. The rest of the operation was the same as the cells. Primary antibodies for Ciao3 (sc‐514078, 1/1000) were obtained from santa, GPX4 (ab125066, 1/2000) and FTL (ab6990, 1/500) from Abcam, xCT (NB300‐318, 1/2000) and FTH1 (NBP1‐31944, 1/500) from Novus, TFR1 (13‐6800, 1/500) from Invitrogen, GAPDH (ab8245, 1/1000) from Abcam. Appropriate secondary antibodies (anti‐rabbit, anti‐mouse and anti‐goat) conjugated to HRP were used (Dako). The density of the brands was quantified by densitometric analysis using Image J. For Co‐IP, the Protein A/G microspheres were washed twice with PBS and prepared into A 50% Protein A/G working solution with PBS. About 1 µg of IgG of the same species as the IP monoclonal antibody and 100 µL of Protein A/G working solution were added to 1 mL of cleavage solution. The mixture was incubated at room temperature for 1 h, centrifuged at 13 000 g for 10 min, and the supernatant was transferred to a new centrifuge tube. This step can remove the non‐specific binding of proteins to immunoglobulins. Then, a certain volume of anti‐precipitation antigen was added, followed by 100 µL Protein A/G working solution to capture the antigen‐antibody complex, which was incubated at 4 °C and shaken overnight. After centrifugation at 13 000 g for 1 min, the precipitate was collected and washed 3 times with pre‐cooled PBS. After the last cleaning, the supernatant was removed by centrifugation and the precipitation was retained. Add 100 µL of 1×loading Buffer for re‐suspension precipitation, take another EP tube, add the same amount of IgG of the same species as the above primary antibody, as a negative control, and immerse in boiling water at 100 °C for 5 min. Before loading, all samples were centrifuged at 4 °C, 13 000 g for 10 min, and then loaded at 20 µL per well.

### Immunohistochemistry and Immunofluorescence of Lung and Mouse Embryo Sections

Cryostat sections were cut from OCT‐embedded lung tissues at 5 µm and mounted on gelatin‐coated histological slides. Slides were thawed at room temperature for 20 min and rehydrated in wash buffer for 10 min. All sections were blocked in 10% goat serum and incubated with primary antibody and Alexa 488, CY3, and CY5‐conjugated secondary antibodies (Thermo Fisher Scientific) for immunofluorescence. Primary antibodies against CIAO3 (NBP1‐83611; 1:200) and CD31 (ab182981; 1:100) were obtained from Novus Biologicals and Abcam, respectively. Primary antibodies against α‐SMA (BM0002; 1:100) and CYP2J2 (ATA27790; 1:500) were purchased from BOSTER Biological Technology and Atagenix, respectively. Primary antibody against Endomucin (GB112648; 1:300), CD34 (GB13584; 1:200), γH_2_AX (GB111841; 1:200), CD31 (GB113151; 1:200) and SMA (GB13044; 1:1000) were obtained from Servicebio. Pictures were obtained using Leica confocal microscopy (TCS SP8). Small pulmonary vessels (< 100 µm diameter) present in a given tissue section (>10 vessels/section) that were not associated with bronchial airways were selected for analysis. Intensity of staining was quantified using Image J (NIH). Degree of pulmonary arteriolar muscularization was assessed in OCT lung sections stained for α‐SMA by calculation of the proportion of fully and partially muscularized peripheral (< 100 µm diameter) pulmonary arterioles, as previously described.^[^ ^34^ ^]^ Endothelial cell marker CD31 and endothelial progenitor cell marker CD34 were used for immunofluorescence detection in whole embryos; DNA damage was detected by γ‐H2AX.

### Ex Vivo Mouse Aortic Ring Assay

The subpackaged matrix glue was melted at 4 °C, and 100 µL was spread in a 48‐well cell culture plate, incubated at 37 °C for 30 min to solidify the matrix glue and create three holes. Wild‐type mice and Ciao3^+/−^ mice were taken, and the free aorta was extracted under sterile conditions after ether anesthesia, and the para‐aortic fat and other tissues were removed. The aorta was carefully divided into an aortic ring with a width of ≈1 mm, and the obtained aortic ring was placed on the solidified matrix glue. 100 µL of melted matrix glue was added to cover the aorta ring and the culture plate was incubated at 37 °C for 30 min. Then, 200 µL of ECM medium containing 5% FBS was added, and the cell were cultured for 4 days. After the culture was completed, photos were taken, and the angiogenesis analysis plug‐in in Image J was used to analyze the acquired images, including the number of blood vessel branches, the total length of blood vessels, and the vessel branches.

### Endomucin and EdU Co‐Staining

For the simultaneous detection of proliferating cells and endothelial cells, co‐staining for EdU (5‐ethynyl‐2′‐deoxyuridine) and Endomucin was performed. Pregnant mice were intraperitoneally injected with EdU (10 mg/kg body weight), and embryos were harvested 6 h later. The embryos were fixed in 4% paraformaldehyde for 2 h at 4 °C, dehydrated through a graded sucrose series, embedded in OCT compound, and cryo‐sectioned at a thickness of 10 µm. The staining procedure was conducted as follows: Tissue sections were permeabilized and blocked with a solution containing 0.5% Triton X‐100 and 10% normal goat serum in PBS for 1 h at room temperature. The EdU detection was carried out first using the Click‐iT EdU Alexa Fluor 488 Imaging Kit (Thermo Fisher Scientific, C10337) according to the manufacturer's instructions. Briefly, sections were incubated with the Click‐iT reaction cocktail for 30 min at room temperature, protected from light. Following thorough washing with PBS, the sections were then incubated overnight at 4 °C with a primary antibody against Endomucin (Mouse Monoclonal [V.7C7], sc‐65495, Santa Cruz Biotechnology) diluted in blocking solution. After washing, the sections were incubated with a secondary antibody (Alexa Fluor 594‐conjugated goat anti‐mouse IgG, Thermo Fisher Scientific) for 1 h at room temperature in the dark. Finally, nuclei were counterstained with DAPI. Slides were mounted with an anti‐fade mounting medium and imaged with a confocal laser‐scanning microscope.

### Behavioral Analyses

Behavioral experiments were conducted on WT and Ciao3^+/−^ male mice. Spontaneous locomotor activity was evaluated using the open‐field paradigm, which allows unrestricted exploration while minimizing external disturbances. During testing, subjects were introduced into a 60 × 60 cm chamber and monitored for 20 min through automated video tracking (Ethovision 6.1, Noldus, Netherlands). Key parameters, including cumulative movement distance, average speed, and zone preference, were systematically quantified.

### Human and Animal Subjects and Ethical Considerations

For patients with pulmonary hypertension with obvious pulmonary vein or pulmonary capillary involvement, the inclusion criteria are: the gold standard was the average pulmonary arterial pressure (mPAP) measured by right cardiac catheter ≥ 25 mmHg; Or echocardiography: Estimation of pulmonary artery pressure from pulmonary valve regurgitation beam spectrum, right atrial regurgitation beam spectrum, and tricuspid regurgitation flow. Exclusion criteria: Pulmonary hypertension caused by congenital heart disease, left ventricular disease, and lung disease and/or hypoxia was excluded. For patients with cerebral small‐vascular disease, the inclusion criteria are: cerebrovascular diseases, including epilepsy, Alzheimer's disease, lacunar cerebral infarction, Binswanger encephalopathy, autosomal dominant cerebral arteriopathy, and amyloidosis cerebrovascular disease with subcortical cerebral infarction and leukoencephalopathy. MRI findings of the head meet the imaging diagnostic criteria of cerebrovascular disease (lacunar cerebral infarction, white matter changes and cerebral microhemorrhage). Exclusion criteria: Imaging changes caused by carbon monoxide poisoning, severe sleep apnea syndrome, brain changes caused by infection and trauma. For patients with systemic lupus erythematosus caused by endothelial cell injury, the clinical symptoms meet the diagnostic criteria revised by the American Rheumatology Society in 1997. Immunological abnormalities include anti‐ds‐DNA antibody positive, anti‐Sm antibody positive, or anti‐phospholipid antibody positive (the latter includes anticardiolipin antibody, lupus anticoagulant positive, or syphilis serum test false positive for at least 6 months), and the levels of vWF, MDA, and GSH and SOD markers of systemic lupus erythematosus endothelial cell injury were increased. The following information was collected by consulting: the enrollment and admission records, electronic medical records, and laboratory examination information: age, sex, smoking (yes/no; have/present), alcohol consumption (yes/no), Body mass index (BMI), echocardiography, and routine biochemical indicators. All clinical data and information were collected in a blinded manner, and data collection, collation, entry, and verification were completed by different personnel. This study strictly adheres to the Helsinki Declaration and has been approved by the Ethics Committee of Zhongnan Hospital of Wuhan University (Ethical Approval: #2020195 and #2025015K).

### NARFL gene tagSNP was Detected by the SNaPShot Method

A total of 49 TagSNPs were selected. To refer to http://grch37.ensembl.org/Homo_sapiens./ Gene/VariationGene /Tabledb=core;g=ENSG00000103245; r = 16:779753‐791329, and 49 tagSNPs were further screened according to mutation type and 10 case‐control pre‐experiments. Finally, seven TagSNPs *rs61112891, rs2071952, rs117952680, rs9928077, rs3752556, rs11248948*, and *chr‐731143*, were selected for in‐depth analysis of large samples. The SNaPshot method was used for SNP detection, and its basic principle was based on the dideoxy termination method in direct DNA sequencing. The difference was that only different fluorescently labeled ddNTPs were used in the PCR reaction. Since the 3 ′end of the designed sequencing primers was close to the SNP site, each sequencing primer can be extended to obtain one or two oligo‐chain products with SNP information markers under the action of polymerase by designing sequencing primers corresponding to different sites. A reaction system can be realized to detect multiple SNP sites. After genome DNA extraction, sample sorting, DNA detection, synthetic primers, PCR amplification, PCR station alkaline phosphatase treatment, and other steps, the ABI 3730 XL sequencer was used for sequencing.

### Statistics

Data were represented as mean±SEM or mean±SD. For cell culture data, 3 independent experiments were performed in triplicate. Animal numbers were calculated to detect a≥20% difference between the means of the experimental and control groups, with 80% power and an SD of 10%. Data normality was confirmed using Shapiro‐Wilk testing. For comparisons between 2 groups, a 2‐tailed Student *t* test was used for normally distributed data. For comparisons among groups, a 1‐way or 2‐way ANOVA was performed. The genotyping results for the SNPs were assessed using the tool VCFtoPed to find the variation data for this gene in the Chinese population (http://grch37.Ensembl.org/Homo_sapiens/Tools/VcftoPed). Mutagenesis was performed using Haploview 4.2 software. Linkage Format was selected. After the data was imported, the check marker interface was displayed, and the Haween balance cutoff value was set to 0.01. Minor allele frequency (MAF) cutoff value was also set to 0.01; For other defaults, click rescore markers, and Eligible SNPs were automatically filtered. According to the number of the selected functional SNPs, manually cancel those non‐functional SNPs. When all selections were complete, click Tagger. For the SNP that checks markers, set the screening criteria. The r^2^ threshold was 0.8, and other parameters were not adjusted. After confirming the parameters, click Run Tagger, and the screening knot will pop up the results. A total of 49 TagSNPs were selected. To refer to http://grch37.ensembl.org/Homo_sapiens./Gene/VariationGene/Tabledb = core;g = ENSG000001 03245; r = 16:779753‐791329, 49 tagSNPs were further screened according to mutation type and 10 case‐control pre‐experiments. Fisher's exact test and Cochran–Armitage trend test were used to assess demographic characteristics and genotype frequency distributions. The association strength was determined via odds ratios (ORs) and 95% confidence intervals (CIs). All genetic models were evaluated for seven tagSNPs. A value of *p*< 0.05 was considered significant.

## Conflict of Interest

The authors declare no conflict of interest.

## Author Contributions

H.H. and F.Z. designed the experiments. H.H. and J.L. collected the data. L.Y., D.Q., B.L., Y.C., X.Z., W.G., and F.W. provided support of clinical samples. All authors have made substantial contributions to the data analyses and manuscript preparation.

## Supporting information



Supporting Information

Supplemental Video 1

Supplemental Video 2

## Data Availability

The data that support the findings of this study are available from the corresponding author upon reasonable request.
