# Supplementary material for: NARFL Knockout Triggers Ferroptosis‐Driven Vascular Endothelial Dysfunction
Source: Adv Sci (Weinh). 2025 Nov 30;13(8):e15580. doi: 10.1002/advs.202415580 (PMC12884811; doi:10.1002/advs.202415580)
Supplement: Supplementary file 1 — Supporting Information [file ADVS-13-e15580-s002.docx]

**Supplemental Figures and Figure Legends**

**NARFL Knockout Triggers Ferroptosis-Driven Vascular Endothelial Dysfunction**

**
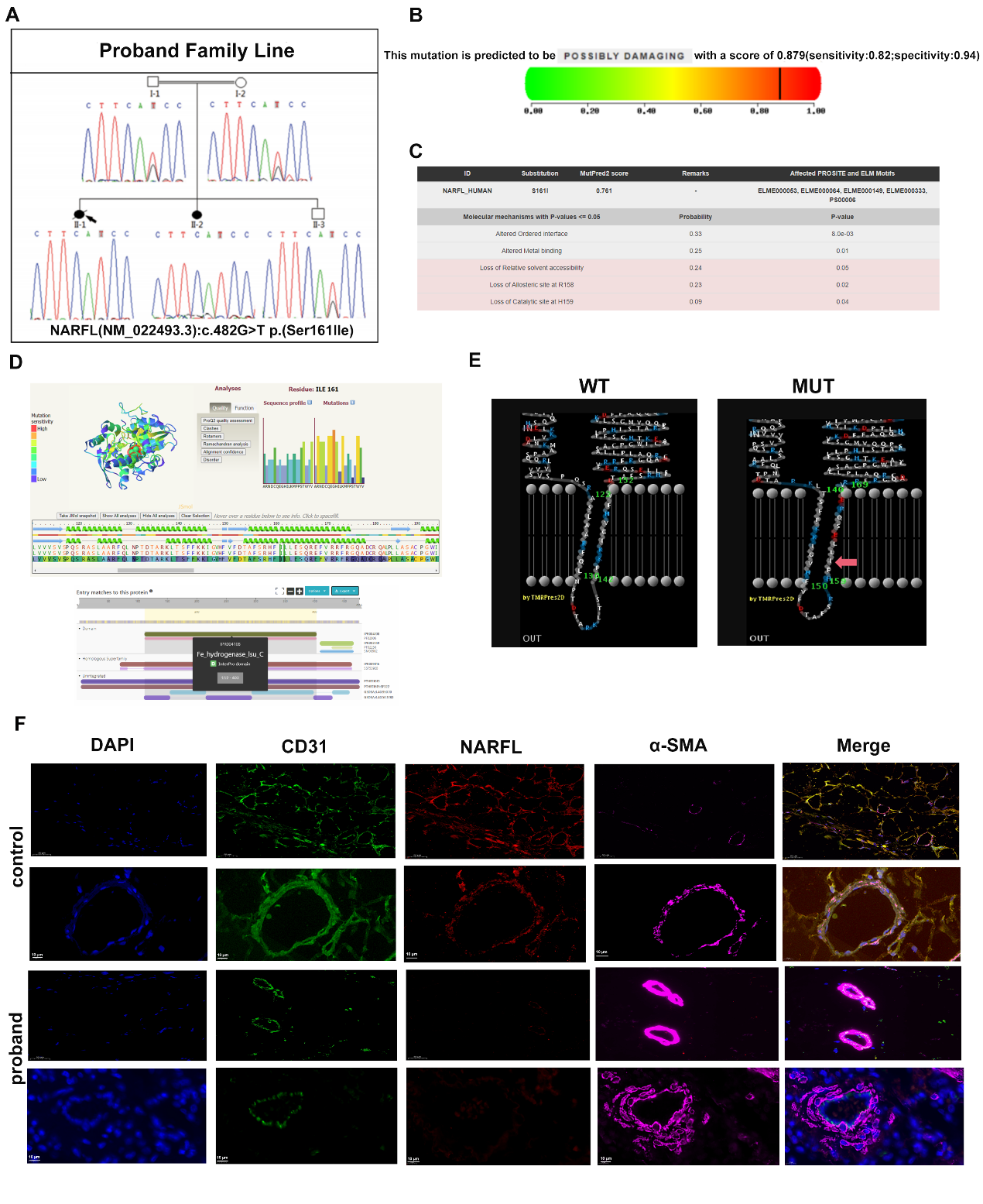
**

**Supplementary Figure 1: Family with pulmonary hypertension secondary to diffuse pulmonary arteriovenous malformation.** (A) The results of sequencing in the family with pulmonary hypertension secondary to diffuse pulmonary arteriovenous malformation: II-1 as the proband and II-2 as the proband sister had homozygous mutation of AGC to ATC in exon 5 of *NARFL* gene (NARFL(NM_022493.3): c.482G>T p. (Ser161Ile)), causing the corresponding amino acid to change from serine (Ser, S) to isoleucine (Ile, I). (B) Polyphen-2 software predicts the pathogenicity of mutations, and its results indicate that the mutation is destructive. (C) The VarSome software predicts the effects of mutation sites, which may alter the metal-binding domain. (D) Function of phyre2 software to predict mutation region: c.482 G>T; The T mutation is in the functional region of the ferric hydrogenase. (E) The PRED-TMBB software predicted that the mutation region changed from a non-transmembrane region to an intra-transmembrane region. (F) Results of fluorescence immunohistochemical staining of the family proband and normal lung tissue. CD31 (green), α-SMA (pink), NARFL (red) DAPI: nucleation; CD31: expressed in endothelial cells; NARFL: expressed in tissue cytoplasm; α-SMA: expressed in myofibroblasts; Merge: Merge all results with a field of view multiple of 100×.


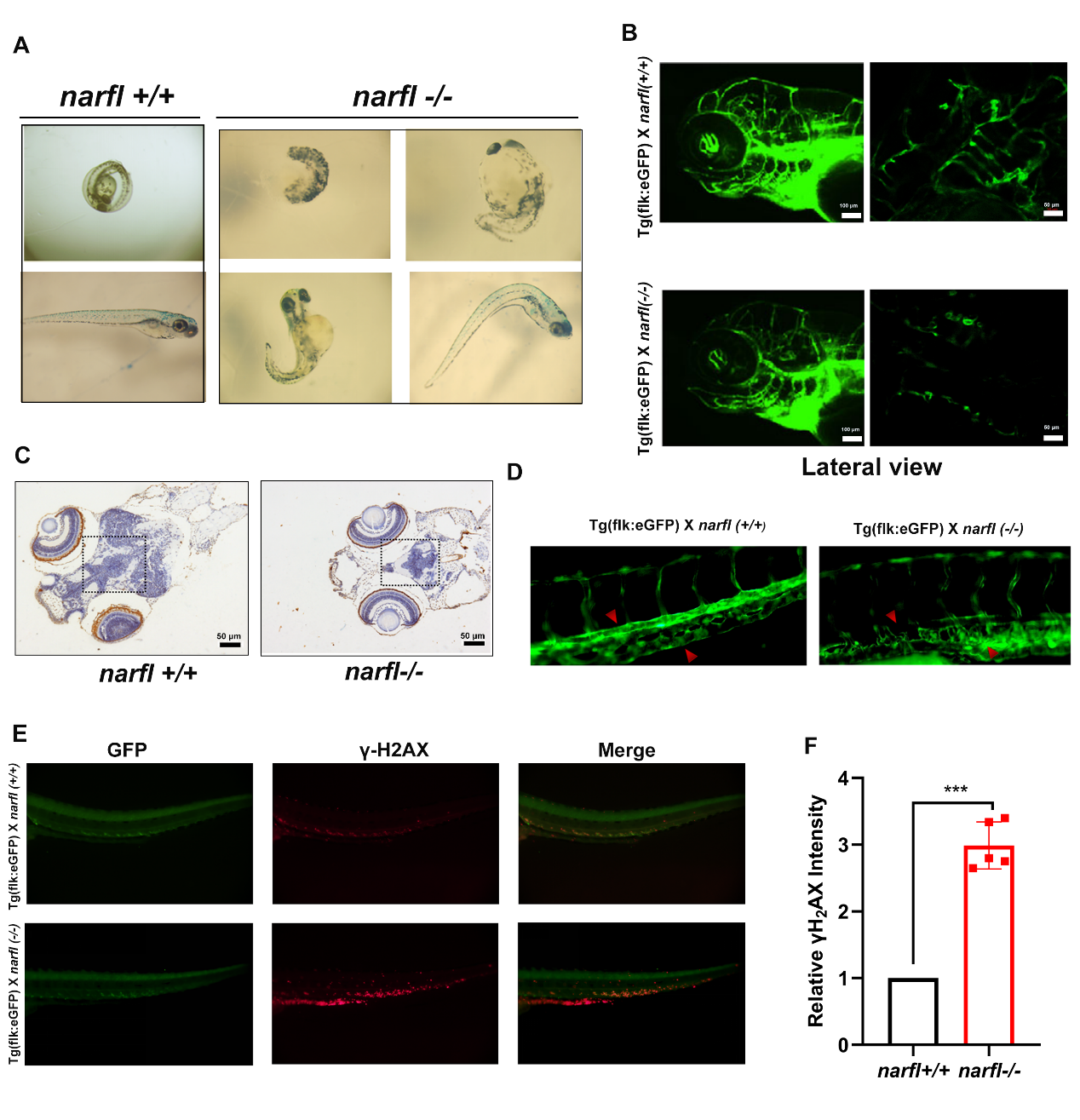


**Supplementary Figure 2. The deletion of narfl gene caused abnormal development and vascular structure of zebrafish.** (A) *narfl^-/-^* Zebrafish showed morphological deformities during development. (B) The blood vessels in the lateral field of zebrafish brains across different genotypes were observed by a fluorescence inverted microscope imaging system. (C) TUNEL staining of the brain of 9-dpf zebrafish with different genotypes in paraffin sections showed that the field of vision was 100×. (D) Fluorescence confocal microscope observation and quantitative analysis of zebrafish vascular segments showed that the dorsal aorta (DA) and posterior cardinal vein (PCV) structure of *narfl^-/-^* Zebrafish were obviously disordered. (E-F) The γH2AX test revealed significant DNA damage in the DA and parts of the PCV, **p*<0.05, ****p*<0.001.


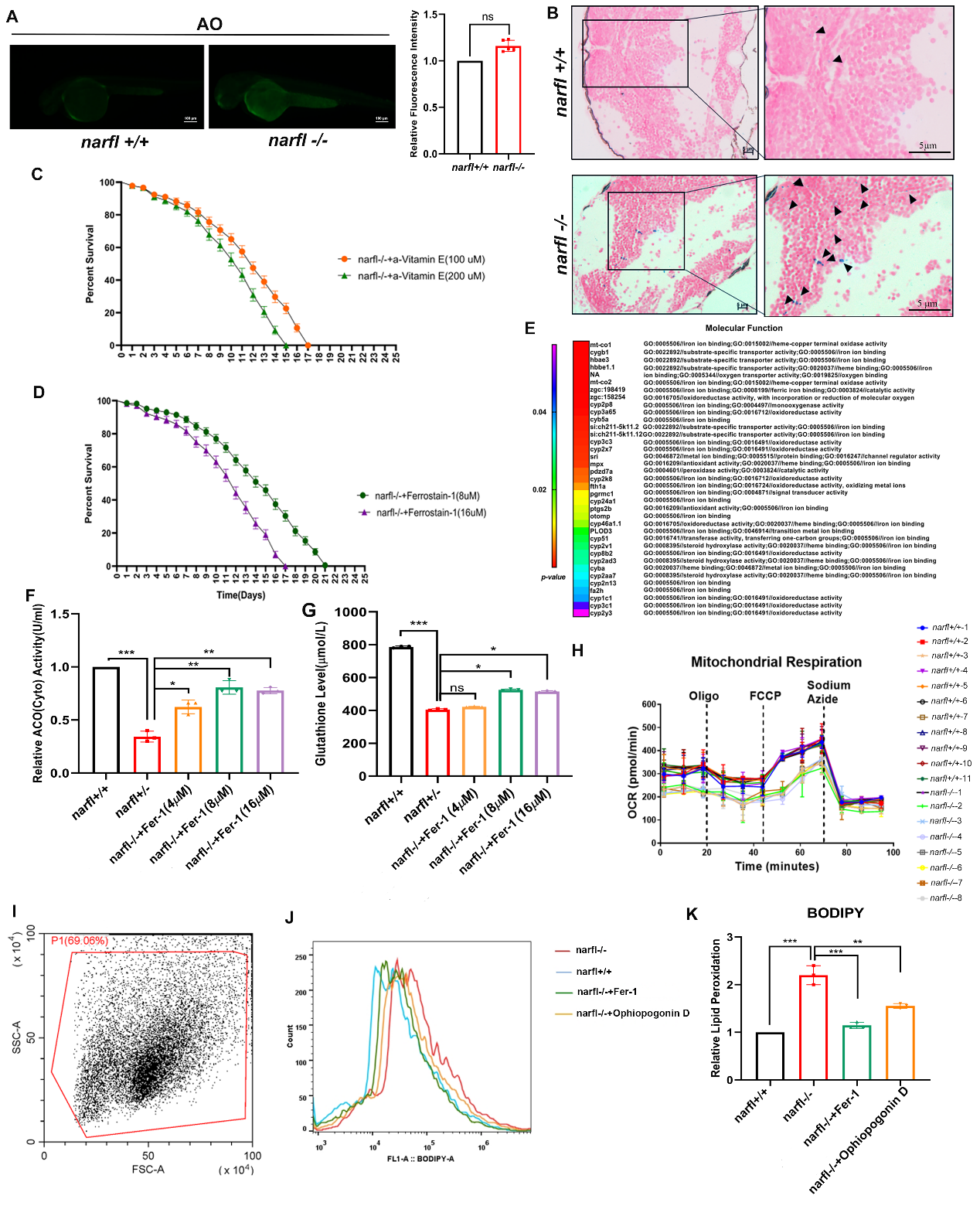


**Supplementary Figure 3. *narfl* gene deletion leads to an increase in oxidative stress, lipid peroxidation and iron levels in zebrafish.** (A) Apoptosis in 5 dpf zebrafish was detected by AO staining; the green fluorescence intensity represented the apoptosis level, and the magnification of the visual field was 200×. (B) The hemosiderin content in the brain of 7 dpf zebrafish was detected by Prussian blue staining. The blue part indicated by the black arrow was 200× and 400× visual field multiple of the positive part, respectively. (C) Ten groups of zebrafish embryos were treated with α-Vitamin E of 100 μM and 200 μM, respectively, the *narfl^-/-^* Zebrafish survival time was extended to 15 dpf after 200 μM α-Vitamin E, and the *narfl^-/-^* survival time was extended to 17 dpf after 100 μM α-Vitamin E. (D) Ten groups of zebrafish embryos were treated with 8 μM and 16 μM Ferrostatin-1, respectively, and *narfl^-/-^* Zebrafish survival time can be extended to 17 dpf after 16μM Ferrostatin-1 treatment, and *narfl^-/-^* Zebrafish survival time can be extended to 21dpf after 8 μM Ferrostatin-1 treatment. (E) List of genes related to iron metabolism and differentially expressed in transcriptomes between wild and *narfl^-/-^* Zebrafish. (F) The cytoplasmic cis-aconitase activity of zebrafish embryos was detected at 5 dpf after Ferrostatin-1 treatment with 4 μM, 8 μM, and 16μM Ferrostatin-1. (G) Zebrafish glutathione levels were detected at 5 dpf after Ferrostatin-1 treatment with 4 μM, 8 μM. and 16 μM Ferrostatin-1. (H) Mitochondrial respiration was detected in 11 wild-type and 8 *narfl^-/-^* Zebrafish. (I-K) The BODIPY levels of wild type and *narfl^-/-^* Zebrafish, treated with Ferrostatin-1 and Ophiopogonin D, were detected by flow cytometry. *p<0.05, **p<0.01, ***p<0.001.


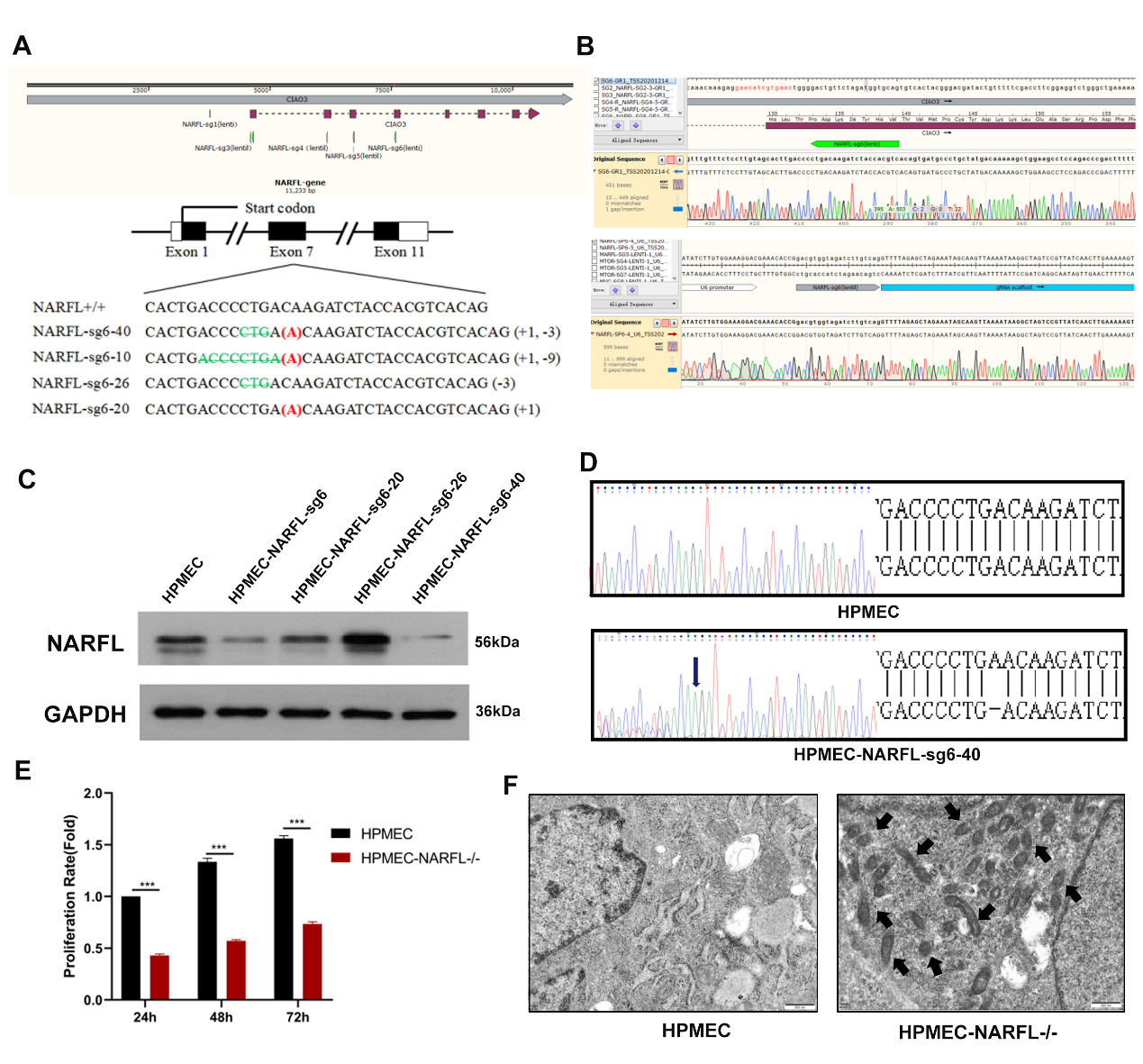


**Supplementary Figure 4. A stable NARFL gene knockdown cell line in HPMEC cells was constructed.** (A)The design location of sgRNA. (B) Plasmid sequencing results. (C) Monoclonal protein western blot results. Compared with wild-type cells, NARFL of HPMEC-*NARFL*-sg6 was significantly down-regulated; NARFL expression in HPMEC-*NARFL*-sg6-20 was not different from that of wild type. NARFL expression in HPMEC-*NARFL* -sg6-26 was higher than that of the wild type. NARFL expression in HPMEC-*NARFL*-sg6-40 was significantly down-regulated compared with the wild type. (D) DNA sequences of HPMEC and HPMEC-*NARFL*-sg6-40. (E) The quantitative results of cell proliferation capacity in two groups were determined by the CCK-8 method for 24h-72h, ****p*<0.001. (F) Abnormal morphology of endothelial mitochondria caused by *NARFL* gene deletion was observed by electron microscope. Black arrows highlight the representative mitochondria with ferroptotic characteristics, consistent with the findings in Figure 3G.


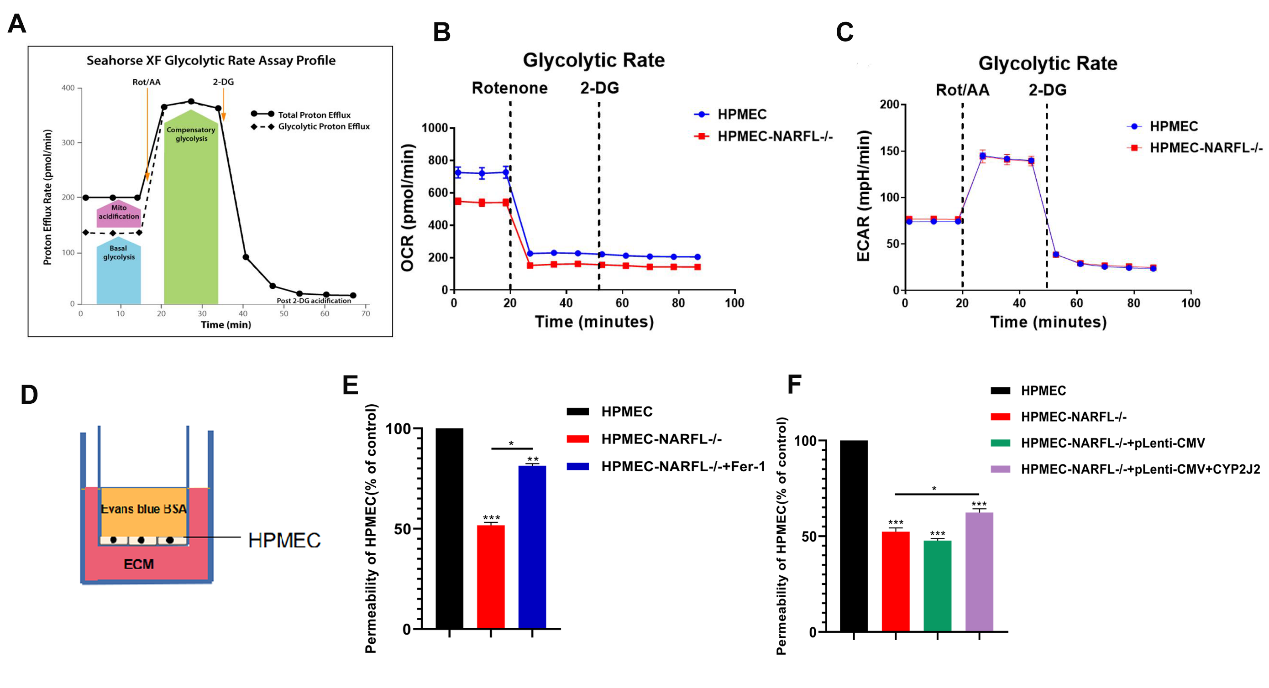


**Supplemental Figure 5. *NARFL* gene deletion does not affect the level of endothelial cell glycolysis but results in endothelial dysfunction.** (A) Model diagram for calculating key parameters of glycolysis in the curve of cell glycolysis rate: basic proton flow rate refers to the number of protons released by cells into the detection solution before adding rotenone/amycin A; mitochondrial acidification rate is the product of mitochondrial oxygen consumption rate and carbon dioxide contribution coefficient; basal glycolysis is the difference between basal proton flow rate and mitochondrial acidification rate; compensatory glycolysis refers to the highest proton outflow rate after adding rotenone/antimycin A; Post 2-DG acidification refers to the lowest value of proton flow rate after adding 2-DG. (B) The glycolysis rate curves for HPMEC and NARFL-mutant HPMEC are shown; blue represents wild-type HPMEC and red represents NARFL-mutant HPMEC. (C) The results of extracellular acidification showed no difference. (D) Diagram of Evans Blue cell penetration experiment, cells were inoculated in a Transwell chamber, and ECM medium was placed in the lower chamber. (E-F) Quantitative results of Evans Blue cell penetration experiment.


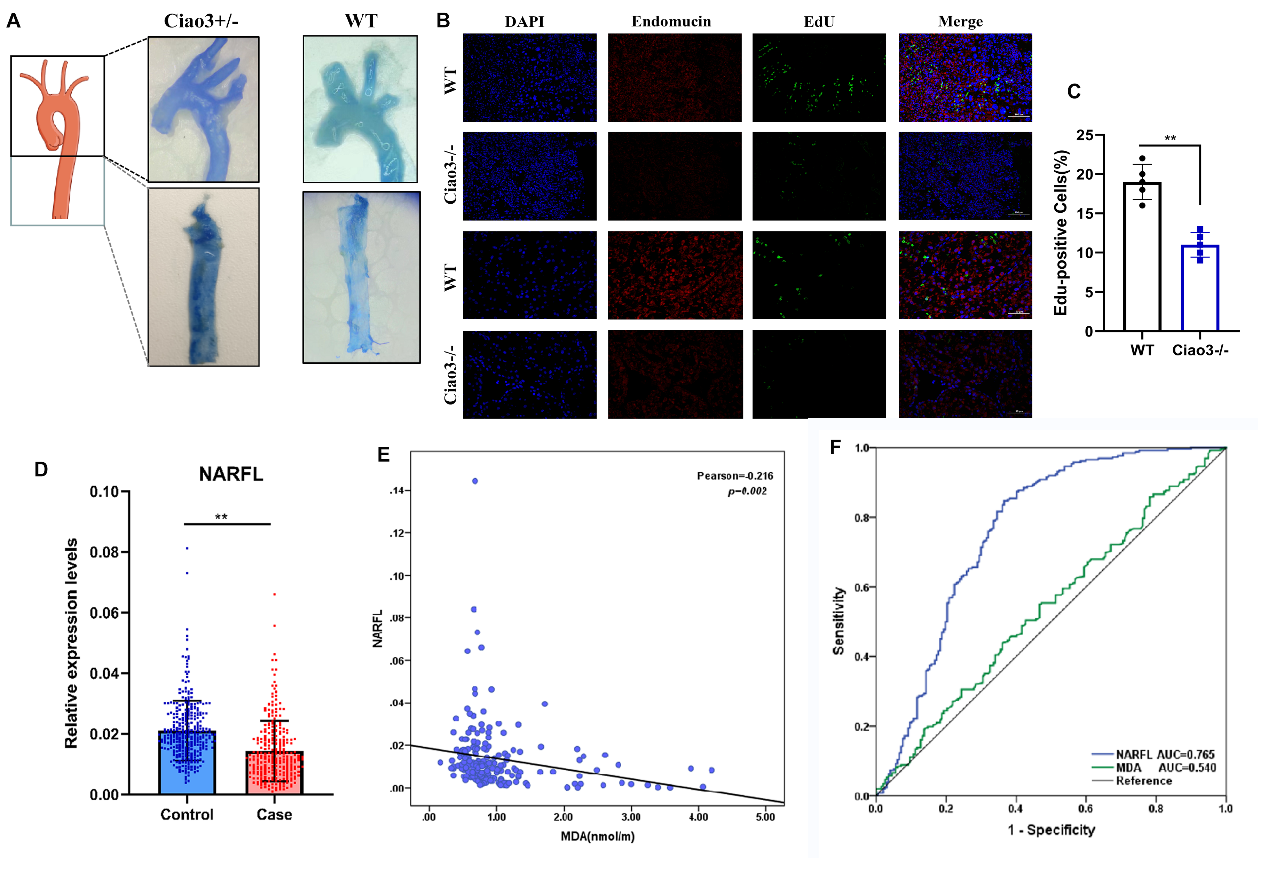


**Supplemental Figure 6. Impairment of Vascular Function in Ciao3 Heterozygous Mice and Ciao3 Polymorphisms are Susceptible Sites of Vascular Endothelial Dysfunction Diseases.** (A) Ciao3^+/-^ mouse aortic arch Evans Blue results; the Evans Blue staining of the aortic arch and cerebrovascular of Ciao3^+/-^ mice was significantly darker than that of the wild type. (B) Representative immunofluorescence images of E12.5 wild-type and Ciao3^-/-^ embryonic tissues co-stained for the endothelial marker Endomucin (red) and the proliferation marker EdU (green). Nuclei are counterstained with DAPI (blue). Upper panels: Low-magnification overview (scale bar = 100 µm). Lower panels: Higher-magnification views of the boxed regions (scale bar = 50 µm). Compared to WT, Ciao3^-/-^ embryos exhibit a poorly formed and disorganized Endomucin-positive vascular network, accompanied by a marked reduction in EdU incorporation, indicating concurrent defects in vascular patterning and endothelial cell proliferation. (C) Statistical significance was determined by one-way ANOVA followed by the Bonferroni test, ***p*<0.01. (D) Quantitative analysis of NARFL expression in a cohort comprising 387 cases and 409 matched controls. Comparative assessment revealed differential expression patterns between the two groups. Significantly lower NARFL expression was observed in cases compared to controls (t (794) = 4.73, *p* < 0.01, d = 0.34, 95% CI: 0.19–0.49). (C) Linear regression was applied to assess the correlation between NARFL and MDA. The result revealed a statistically significant negative association between NARFL and MDA (r = -0.216, *p* = 0.002). (D) The Receiver operator characteristic (ROC) analysis was conducted between the MDA level and NARFL expression level in the disease population with tagSNP difference in the case group, and the results showed that NARFL expression level was more effective at distinguishing the population with tagSNP difference. The Area under the curve (AUC) of NARFL is 0.765, while the AUC of MDA is 0.540, lower than that of NARFL. Data are represented as mean±SEM. Values were obtained by unpaired Student’s t-test, not significant; **p* < 0.05; ** *p* < 0.01; *** *p* < 0.001.

**HPMEC-WT.mp4 and HPMEC-MUT.mp4**: Compared to wild-type HPMEC cells, NARFL-mutant cells demonstrated progressive atrophy, localized membrane rupture, a granular cytoplasmic appearance, and no significant nuclear fragmentation over time.

**Zebra fish behavious video 1:** narfl-deficient zebrafish exhibited circular swimming behavior and marked body rigidity.

**Zebra fish behavious video 2:** narfl-deficient zebrafish exhibited fast circular swimming behavior.

**Zebra fish behavious video 3:** narfl-deficient zebrafish exhibited burst swimming behavior accompanied by a loss of directional control.

**Zebra fish behavious video 4:** narfl-deficient zebrafish exhibited spiral swimming behavior accompanied by generalized clonic convulsions.
